# Structural Basis for Fe–S Cluster Assembly and tRNA Thiolation Mediated by IscS Protein–Protein Interactions

**DOI:** 10.1371/journal.pbio.1000354

**Published:** 2010-04-13

**Authors:** Rong Shi, Ariane Proteau, Magda Villarroya, Ismaïl Moukadiri, Linhua Zhang, Jean-François Trempe, Allan Matte, M. Eugenia Armengod, Miroslaw Cygler

**Affiliations:** 1 Department of Biochemistry, McGill University, Montréal, Québec, Canada; 2 Laboratorio de Genética Molecular, Centro de Investigación Príncipe Felipe, Valencia, Spain; 3 Biotechnology Research Institute, Montréal, Québec, Canada; Brandeis University, United States of America

## Abstract

The cysteine desulfurase IscS is a highly conserved master enzyme initiating sulfur transfer via persulfide to a range of acceptor proteins involved in Fe-S cluster assembly, tRNA modifications, and sulfur-containing cofactor biosynthesis. Several IscS-interacting partners including IscU, a scaffold for Fe-S cluster assembly; TusA, the first member of a sulfur relay leading to sulfur incorporation into the wobble uridine of several tRNAs; ThiI, involved in tRNA modification and thiamine biosynthesis; and rhodanese RhdA are sulfur acceptors. Other proteins, such as CyaY/frataxin and IscX, also bind to IscS, but their functional roles are not directly related to sulfur transfer. We have determined the crystal structures of IscS-IscU and IscS-TusA complexes providing the first insight into their different modes of binding and the mechanism of sulfur transfer. Exhaustive mutational analysis of the IscS surface allowed us to map the binding sites of various partner proteins and to determine the functional and biochemical role of selected IscS and TusA residues. IscS interacts with its partners through an extensive surface area centered on the active site Cys328. The structures indicate that the acceptor proteins approach Cys328 from different directions and suggest that the conformational plasticity of a long loop containing this cysteine is essential for the ability of IscS to transfer sulfur to multiple acceptor proteins. The sulfur acceptors can only bind to IscS one at a time, while frataxin and IscX can form a ternary complex with IscU and IscS. Our data support the role of frataxin as an iron donor for IscU to form the Fe-S clusters.

## Abstract 2

Crystal structures reveal how distinct sites on the cysteine desulfurase IscS bind two different sulfur-acceptor proteins, IscU and TusA, to transfer sulfur atoms for iron-sulfur cluster biosynthesis and tRNA thiolation.

## Author Summary

Sulfur is incorporated into the backbone of almost all proteins in the form of the amino acids cysteine and methionine. In some proteins, sulfur is also present as iron–sulfur clusters, sulfur-containing vitamins, and cofactors. What's more, sulfur is important in the structure of tRNAs, which are crucial for translation of the genetic code from messenger RNA for protein synthesis. The biosynthetic pathways for assembly of these sulfur-containing molecules are generally well known, but the molecular details of how sulfur is delivered from protein to protein are less well understood. In bacteria, one of three pathways for sulfur delivery is the *isc* (iron-sulfur clusters) system. First, an enzyme called IscS extracts sulfur atoms from cysteine. This versatile enzyme can then interact with several proteins to deliver sulfur to various pathways that make iron–sulfur clusters or transfer sulfur to cofactors and tRNAs. This study describes in atomic detail precisely how IscS binds in a specific and yet distinct way to two different proteins: IscU (a scaffold protein for iron–sulfur cluster formation) and TusA (which delivers sulfur for tRNA modification). Furthermore, by introducing mutations into IscS, we have identified the region on the surface of this protein that is involved in binding its target proteins. These findings provide a molecular view of the protein–protein interactions involved in sulfur transfer and advance our understanding of how sulfur is delivered from one protein to another during biosynthesis of iron–sulfur clusters.

## Introduction

Sulfur is a critical element in all living cells, incorporated into proteins not only in the form of cysteine and methionine but also as iron-sulfur clusters, sulfur-containing cofactors and vitamins, and into RNA through a variety of modifications [1],[2]. Delivery of sulfur for these various biosynthetic pathways is a complex process, involving successive transfers of sulfur as persulfide between multiple proteins, many of which are highly conserved across species. Three distinct systems have been identified for the assembly of iron-sulfur clusters: *isc*, *nif*, and *suf* (reviewed in [1],[3]–[5]). The *isc* (iron-sulfur clusters) system participates constitutively in general-purpose iron-sulfur cluster assembly and in transfer of sulfur to several cofactors and tRNAs. The *nif* (nitrogen fixation) system is involved in iron-sulfur cluster assembly required for the maturation of nitrogenase [6], while the *suf* (sulfur mobilization) system plays a role during oxidative stress or iron starvation. The initial step in each system is performed by a specific cysteine desulfurase, IscS [7], NifS [8], or SufS (previously CsdB, [9]), respectively, forming the initial persulfide.

**Figure 1:**
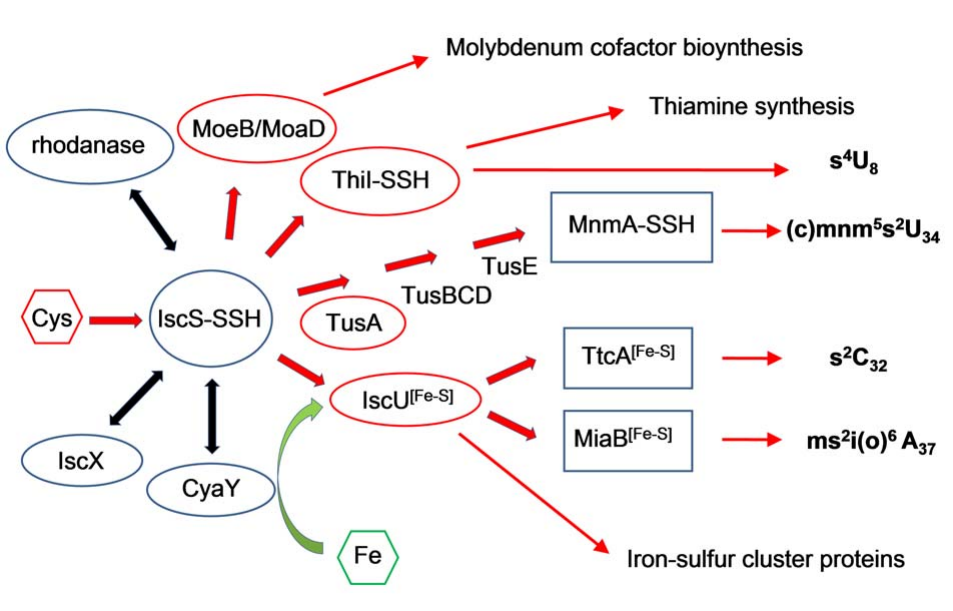
Network of protein-protein interactions involving IscS. IscS initiates intracellular sulfur trafficking, delivering the sulfur to several sulfur-accepting proteins such as IscU, ThiI, TusA, and MoaD/MoeB that commit the sulfur to different metabolic pathways. IscU is the primary scaffold for assembly of Fe-S clusters. Frataxin/CyaY has been postulated as an Fe chaperone, an Fe donor for Fe-S cluster assembly, or a regulator of Fe-S cluster formation. In the schematic, sulfur delivering is indicated by red arrows and IscS-interacting proteins are framed by ovals (red, in sulfur accepting proteins).

IscS is a highly conserved, widely distributed pyridoxal-5′-phosphate (PLP)-dependent enzyme [7],[10], with 60% sequence identity between the enzyme from *Escherichia coli* and its human homolog, NFS1. It initiates intracellular sulfur trafficking, delivering the sulfur to several sulfur-accepting proteins such as IscU, ThiI, TusA, and MoaD/MoeB that commit the sulfur to different metabolic pathways, including iron-sulfur cluster assembly, thiamine and biotin synthesis, tRNA modifications, or molybdopterin biosynthesis [2],[3],[11]. IscU is the primary scaffold for assembly of Fe-S clusters [12] that are required by iron-sulfur proteins. In addition to these sulfur acceptors, IscS interacts with several other proteins, including CyaY, a bacterial homolog of human frataxin [13],[14]; IscX, a possible adaptor protein whose exact function is as yet unknown [15],[16]; and rhodanese RhdA [17]. Frataxin/CyaY has been postulated as an Fe chaperone [18], an Fe donor for Fe-S cluster assembly [13],[19],[20], or a regulator of Fe-S cluster formation [14]. The network of known IscS protein interactions is shown in Figure 1.

Thiolated nucleotides are found in several tRNAs. In *E. coli* and *Salmonella enterica* serovar Typhimurium, these are s^4^U8, s^2^C32, ms^2^i(o)^6^A37, and (c)mnm^5^s^2^U34, which, with the exception of s^4^U8, are located within the anticodon loop and are crucial for proper mRNA decoding [21]. The base thiolations are mediated by several acceptor proteins, falling into two distinct pathways [21]. In the iron-sulfur cluster independent pathway, direct transfer of sulfur from IscS to the acceptor ThiI leads to the s^4^U8 modification [22], while transfer to TusA results in the (c)mnm^5^s^2^U34 modification [23]. ThiI also participates in thiamine biosynthesis [24]. The second pathway proceeds through the formation of an iron-sulfur cluster and is dependent on the IscU acceptor protein. The enzymes TtcA and MiaB accept sulfur from IscU [3] and are responsible for the s^2^C32 [25] and ms^2^i(o)^6^A37 modification [26], respectively. The unique tRNA thiolation pattern associated with sulfur transfer from IscS to TusA, IscU or ThiI provides a convenient readout system to assess the in vivo effects of IscS mutations on its interaction with these proteins.

The proteins involved in sulfur utilization have been extensively studied both functionally and structurally. Structures of IscS [27], the sulfur acceptor proteins TusA [28], ThiI [29], IscU [30],[31], rhodanese [32], and the modulators human frataxin [33],[34] and its bacterial homologue CyaY [35],[36], as well as IscX [16],[37] have been determined by X-ray crystallography or NMR. All of these proteins adopt different folds and the acceptor proteins receive sulfur from IscS by molecular mechanisms that are not fully understood.

Despite this wealth of structural information, the question of how IscS is able to communicate with such a broad spectrum of proteins and deliver sulfur to a wide range of structurally divergent partners is unresolved as no structural information on its complex(es) with binding partner(s) is presently known. To begin addressing this question, we have determined the crystal structure of the IscS-TusA and the IscS-IscU complexes, which reveal different modes of binding of these proteins and provide a framework for understanding sulfur transfer from IscS. Further, we performed extensive mutagenesis of the IscS surface followed by in vitro (pull-down) and in vivo (tRNA complementation assay) studies to map the interface with ThiI, CyaY/frataxin and IscX. Competition for binding to IscS by its various partners has been explored by three-way pull-down experiments.

## Results

### Molecular Interfaces of the IscS-TusA and IscS-IscU Complexes

**Figure 2:**
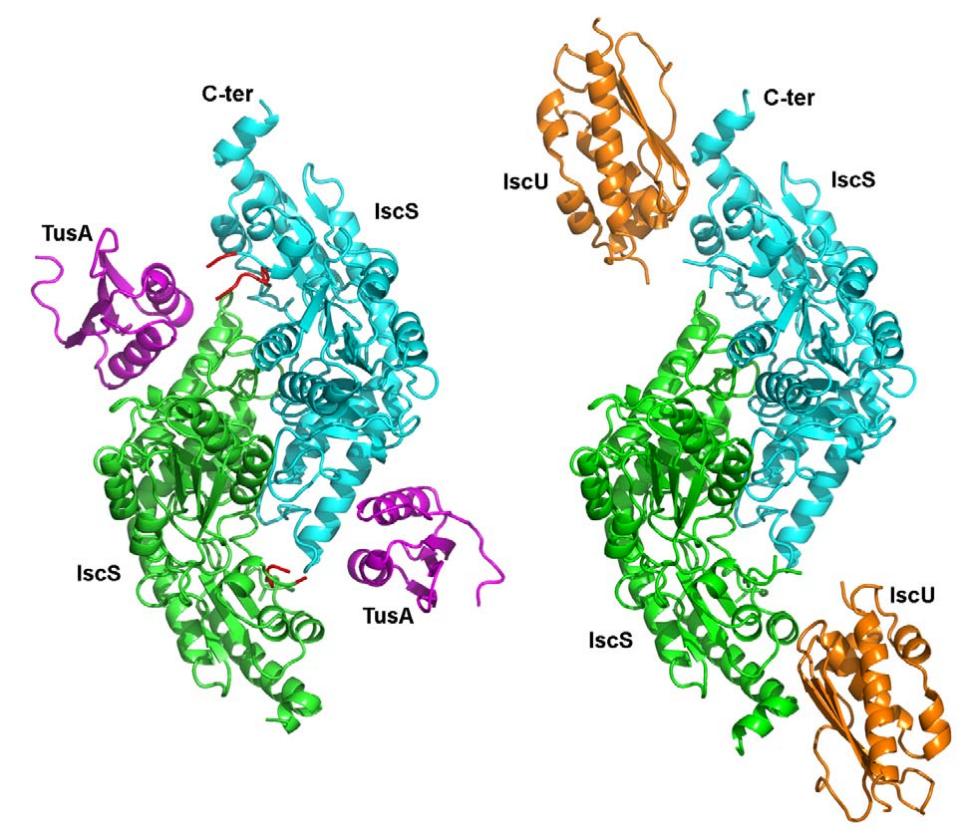
Crystal structure of IscS complexes. Cartoon representation of the IscS-TusA and IscS-IscU heterotetramers. The IscS subunits are colored cyan and green, TusA is magenta and IscU is orange. The Cys328 containing loops are red.

We have crystallized and determined the structures of the *E. coli* IscS-TusA and IscS-IscU complexes at 2.45 Å and 3.0 Å resolution, respectively (Figure 2 and Table 1). The atomic structures of these complexes provide a detailed description of two different protein binding sites on the IscS surface.

**Table 1:** X-ray data collection and refinement statistics.

Dataset	IscS-TusA Form 1	IscS-TusA Form 2	IscS-IscU	IscS
Space group	*P*2_1_2_1_2_1_	*C*222_1_	*P*6_1_22	P212121
a, b, c (Å)	72.3, 106.5, 122.1	72.9, 131.4, 106.4	77.6, 77.6, 356.0	74.8, 99.2, 118.1
Wavelength(Å)	0.9793	0.9793	0.9793	0.9793
Resolution (Å)	50–2.45 (2.54–2.45)	50–2.45 (2.54–2.45)	50–3.0 (3.11–3.0)	50–2.05 (2.12–2.05)
Observed hkl	204,100	135,300	190,247	297,352
Unique hkl	34,585	17,507	11,488	52,419
Redundancy	5.9	7.7	16.6	5.7
Completeness (%)	96.4 (79.0)	91.3 (58.3)	83.4 (36.5)	93.5 (66.7)
Rsym^a^	0.072 (0.365)	0.077 (0.385)	0.093 (0.285)	0.068 (0.419)
I/(σI)	13.1 (2.8)	15.5 (3.0)	12.1 (4.4)	14.6 (2.3)
Wilson B (Å^2^)	51.1	56.0	72.3	32.9
Rwork^b^ (# hkl)	0.222 (32,712)	0.207 (16,569)	0.225 (10,917)	0.198 (49,635)
Rfree (# hkl)	0.240 (1,722)	0.249 (883)	0.269 (555)	0.239 (2,683)
**B-factor(Å^2^) (# atoms)**				
Protein	49.1 (7,205)	82.4 (3,619)	65.9 (3,938)	38.8 (6,155)
Solvent	43.5 (213)	55.9 (46)	57.0 (13)	42.8 (310)
Ligands	72.7 (30)	103.4 (15)	60.6 (15)	47.7 (30)
**Ramachandran**				
Allowed (%)	100	99.6	98.5	99.7
Generous (%)	0	0.2	1.1	0
Disallowed (%)	0	0.2	0.4	0.3
R.m.s. deviation				
Bonds (Å)	0.004	0.007	0.004	0.012
Angles (°)	0.68	1.01	0.65	1.41
PDB code	3LVJ	3LVK	3LVL	3LVM

a
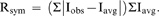

b
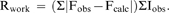

IscS is composed of two domains [27]. The small domain (residues 1–15 and 264–404) contains the critical active site cysteine Cys328. The large domain (residues 16–263) harbours the PLP cofactor and the cysteine substrate-binding pocket. Dimerization of IscS predominantly involves residues from the large domain. Easily recognizable electron density in our structures indicated the presence of the PLP cofactor as an internal aldimine covalently bound to Lys206, as previously observed [27]. TusA has a compact two-layered α/β-sandwich structure with a central four-stranded mixed β-sheet having the connectivity β1↑β2↑β4↓β3↑ and two α-helices [28]. IscU is a two-layered α/β sandwich with a core three-stranded β-sheet and bundle of five α-helices [31].

**Figure 3:**
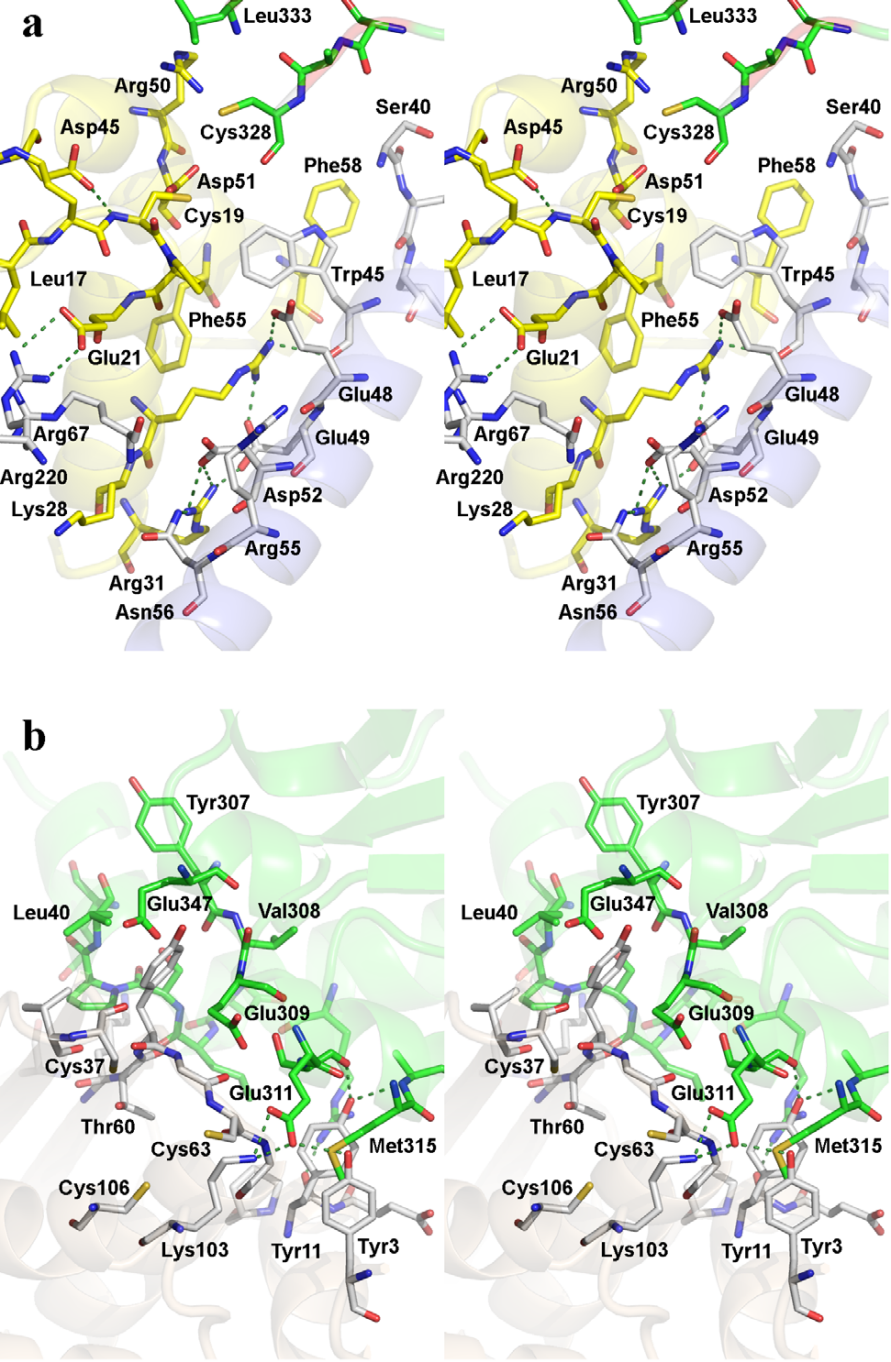
Interface between IscS and TusA or IscU. (A) IscS-TusA, IscS (gray carbons), and TusA (yellow carbons). The Cys328^IscS^ and Leu333^IscS^ from the second subunit are shown with green carbons. The IscS residues in between are disordered. The conserved Asp45^TusA^ and Asp51^TusA^ are shown explicitly in stick mode. Hydrogen bonds are marked as dashed lines. Salt bridges Arg27^TusA^…Glu49^IscS^…Arg31^TusA^…Asp52^IscS^ in the center of the interface and Glu21^TusA^…Arg220^IscS^ at the periphery are explicitly shown; (B) IscS-IscU: IscU, gray carbons. The residues displayed are within 3.7 Å of its binding partner.

The IscS-TusA complex crystallized in two forms with identical heterotetramers consisting of an IscS dimer and two TusA molecules. The distance between the two TusA monomers exceeds 40 Å (Figure 2). TusA interacts with the large domain of one IscS subunit within the dimer, with the exception of the tip of the loop containing the essential Cys328 of IscS, which comes from the other subunit (Figure 2). This persulfide-carrying Cys328^IscS^ is juxtaposed against the acceptor cysteine of TusA, Cys19^TusA^, with only ∼4 Å separating their S atoms. Most of the IscS residues involved in the interaction with TusA are located on the outside face of a six-turn helix α2, the N-terminus of strand β2, the C-terminus of the neighbouring strand β9, and the following loop β9/α7 (Figure 3A and Figure S1). Electron density for the interface residues is shown in Figure S2A. The residues of TusA contacting IscS are located on two α-helices (α1^TusA^ and α2^TusA^), which are nearly perpendicular to helix α2^IscS^. Formation of the complex buries the α-helical layer of TusA and leaves its β-sheet layer exposed to the solvent. Approximately 710 Å^2^ of the molecular surface of each binding partner is buried, corresponding to ∼16% of the total TusA surface area. The interface involves van der Waals contacts, polar and hydrogen bond interactions, and salt bridges (Figure 3A). The main van der Waals contacts are provided by TusA Met24^TusA^, Met25^TusA^ (α1^TusA^), Phe55^TusA^, Phe58^TusA^, Met59^TusA^ (α2^TusA^) and IscS Trp45^IscS^ (stacking with Phe58^TusA^), and the aliphatic portions of Arg55^IscS^ and Arg237^IscS^.

As established previously [38], the IscS-IscU complex is also a heterotetramer. IscU binds near the C-terminus of IscS, forming a very elongated S-shaped heterotetrameric protein complex 150 Å long and 65 Å wide (Figure 2). The IscU is in its apo form, with no evidence of a bound Fe-S cluster. IscU makes contacts with helix α8^IscS^ (Glu309-Ala316), helical turn α10^IscS^ (Glu347), the end of helix α11^IscS^, and the C-terminal helix α12^IscS^ (Arg379-Lys391). The importance of the latter contact is emphasized by the lack of binding of IscU to IscS(Δ376-404) [39]. The contacts on IscU include Tyr3 and Tyr11 (N-terminus), Gly38, Val40 and Lys42 (β2^IscU^), Lys59-Gly64 (β3^IscU^), and Lys103 (Figure 3B, electron density in Figure S2B). The IscU surface area buried upon complex formation is ∼790 Å^2^. The bound IscU projects its most conserved surface containing three conserved cysteines (Figure S3) toward the IscS loop that carries Cys328. The distance between the modeled Cys328^IscS^ and any cysteine of IscU in our structure is greater than ∼12 Å, implying that a conformational change must accompany sulfur transfer (Figure S4). The contacts provided by the N-terminus and helix α1^IscU^ (Glu5-Glu12) are critical for the formation of the cognate complex, as confirmed by a partial loss of in vitro binding of IscU(Δ1-7) to IscS and a complete loss of binding of IscU(Δ1-12) (Table 2 and Figure S5A). We constructed several IscU point mutants of residues on loops facing IscS to verify the interface observed in the IscS-IscU structure. Only the charge reversal mutant K103^IscU^E located within the interface and pointing toward IscS disrupted the complex (Table 2). Removing the sidechain of another residue located at the interface, Tyr11 (Y11A), had no significant effect on binding as this was not a disruptive mutation. Finally, the charge removal/reversal mutants E5L, D9R, and E98R located outside the observed interface had no effect on complex formation.

**Table 2:** Interaction between wild-type IscS and IscU mutants measured in vitro by pull-down (yes, binding observed; no, no binding).

IscU Mutants	Wt IscS, In Vitro Pull-Down
E5L	Yes
D9R	Yes
Y11A	Yes
E98R	Yes
K103E	No
Δ1–6	Low
Δ1–12	No
Δ1–17	No

**Figure 4:**
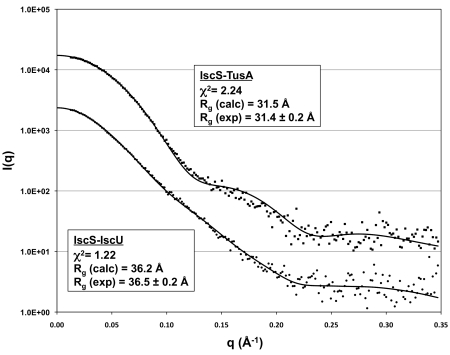
Small-angle X-ray scattering (SAXS) analysis of the complexes formed by IscS in solution. Scattering data (desmeared, merged, and binned) are shown as squares and circles for the IscS-TusA and IscS-IscU complexes, respectively. The predicted scattering profiles calculated in CRYSOL from atomic coordinates are shown as plain black lines. The profiles were offset on the vertical axis for clarity.

To determine if the IscS-TusA and IscS-IscU complexes existing in solution are the same as the heterotetramers observed in the crystal structures, we performed small angle X-ray scattering (SAXS) experiments. The scattering curve obtained for the IscS-TusA complex at a protein concentration of 22 mg/ml fit very well (χ^2^ = 2.24) to the intensity profile calculated from the crystal structure of the complex (Figure 4), indicating that the crystal and solution structures represent the same biological unit. Similarly, the data for the IscS-IscU complex are in excellent agreement (χ^2^ = 1.22) with the very elongated structure observed in the crystal (Figure 4).

### Structural Rearrangements upon Complex Formation

**Figure 5:**
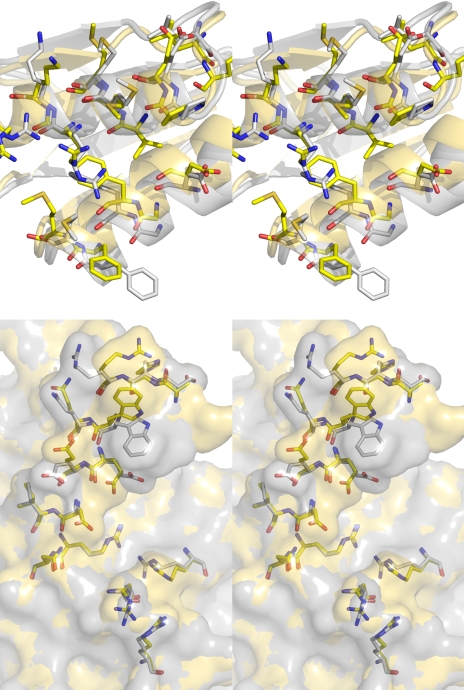
Split open IscS-TusA interface (gray) with superposed uncomplexed proteins (yellow). Above, TusA with secondary structure elements; below, IscS with semitransparent molecular surface. The reorientation of multiple sidechains creates better shape complementarity between the contacting molecular surfaces.

Formation of the IscS-TusA or IscS-IscU complexes is associated with only minor conformational changes in the IscS dimer, predominantly of surface sidechains. The root-mean-square deviation (rmsd) between free (PDB code 1P3W) and TusA-bound IscS is ∼0.4 Å for the corresponding ∼380 Cα atoms. Nevertheless, sidechain reorientation results in a significant change in the shape of the IscS binding surface and improves surface complementarity to TusA (Figure 5). There is no change in the active site pocket containing the PLP cofactor.

The TusA molecules in the complex show larger structural deviations from the individual TusA structures as determined by NMR spectroscopy (PDB code 1DCJ, [28]) (rmsd of ∼1.3 Å for all Cα atoms), corresponding to a ∼2.5 Å shift of helix α2^TusA^ away from α1^TusA^ along the surface of the β-sheet, accompanied by a small ∼15° rotation of this helix along its axis.

Upon binding of IscU to IscS, the major structural change in IscU relative to the solution structures of IscU from *H. influenza* [30], *B. subtilis* (PDB code 1XJS), and mouse (PDB code 1WFZ) involves ordering of the ∼25 N-terminal residues and folding of Glu5-Glu12 into an α-helix, thereby providing crucial contacts with IscS. This segment is largely disordered in all solution structures of IscU and the N-terminus assumes different conformations in three independent molecules in the crystal structure of *Aquifex aeolicus* IscU [31]. The rmsd between *E. coli* IscU and *Aquifex aeolicus* IscU is ∼1.3–1.6 Å for the ordered ∼100 Cα atoms segment.

### Mapping the Protein-Protein Interacting Surface of IscS

**Figure 6:**
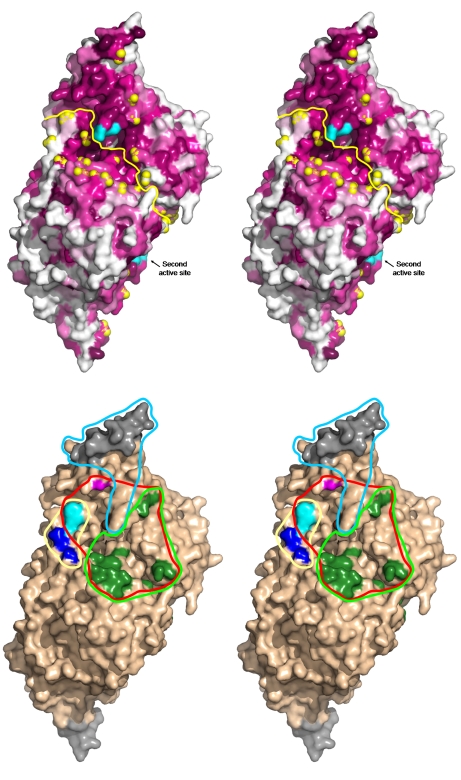
Protein binding surface of the IscS dimer. (A) Residue conservation pattern on the surface of the IscS dimer. The view is toward the active site Cys328. The yellow line indicates the dimer interface and the yellow spheres mark the tips of the residues that have been mutated. The level of conservation of surface residues is marked in shades of burgundy (dark, high conservation; white, highly variable). The residues Cys328-Ser336 are colored cyan. (B) location of mutations affecting interaction with acceptor proteins: IscU, gray; TusA, blue; ThiI, in magenta; TusA/ThiI, cyan; CyaY/IscX/ThiI, dark green. The C-terminal residues 376–404 colored gray at the top-right are missing in the Δ376–404 deletion mutant. The footprint of IscU is marked by light blue line: TusA, yellow line; ThiI, red line; and IscX, CyaY, green line.

The structures of IscS-IscU and IscS-TusA identified non-overlapping IscS surfaces (with the potential exception of the disordered tip of Cys328^IscS^ loop) interacting with IscU and TusA. However, IscS also interacts with several other proteins and we aimed to identify the “active” surface of IscS. We first analyzed the pattern of surface residue conservation using the CONSURF server (http://consurf.tau.ac.il/; [40]). The conserved residues form a large, contiguous molecular surface extending across the dimer interface and centered on the active site Cys328 (Figure 6A). The extent of the conserved surface suggests that a substantially larger surface area than that observed for the IscS-IscU and IscS-TusA complexes is utilized for binding all protein partners.

To further characterize the IscS binding surface we expressed and purified three other proteins in addition to IscU and TusA, namely the sulfur acceptor ThiI, a modulator frataxin/CyaY, and IscX from the *isc* operon. All of these proteins have previously been shown to bind to IscS. The IscS utilized in this study had not been charged with the persulfide group. Nevertheless, all IscS partners formed stable complexes, indicating that Cys328 does not need to be present in the persulfide form for protein-protein binding (see below).

**Figure 7:**
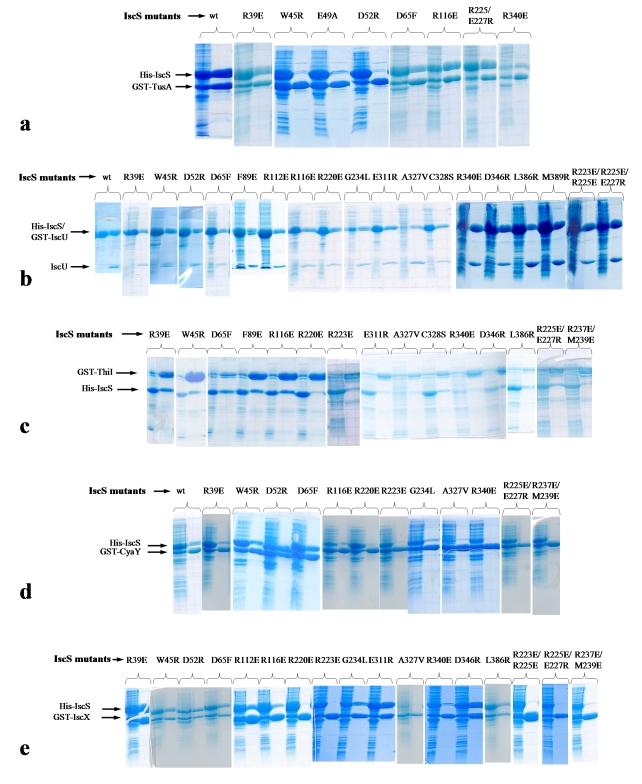
Interactions of IscS mutants with binding partners determined by in vitro pull-downs. Only interactions of representative mutants are shown. The IscS is His-tagged, the partners are GST-tagged, and the mixture was loaded on the glutathione Sepharose, the beads washed, and analyzed by SDS-PAGE. The mutations are indicated above the lanes. Two lanes are shown for each mutant: left shown the mixture loaded on the column; right, proteins retained on the column. (A) TusA; (B) IscU. His-IscS and Gst-IscU appear at the same place on the SDS gel (left lane). To distinguish between them the proteins were released from the beads by TEV protease cleavage of the GST and elution of His-IscS and untagged IscU. Only A327V show a small decrease in the IscS/IscU ratio; (C) ThiI; (D) CyaY; (E) IscX.

To experimentally map the IscS interacting surface, we created a series of IscS point mutations distributed across the entire conserved surface (Figure 6A and Table 3). The mutations were designed to invert the polar or nonpolar character of a specific residue, or replace a smaller sidechain by a larger one. For in vitro pull-down experiments, all mutant proteins were expressed and purified following the same protocol as for wild-type IscS and showed similar behaviour during purification. IscS mutations that abrogated interaction with wild-type TusA, W45^IscS^R, E49^IscS^A, D52^IscS^R (Figure 7A), D52^IscS^Y, and D52^IscS^M (unpublished data) involved tightly clustered residues located on the side of helix α2^IscS^, in excellent agreement with the crystal structure. A significant contribution of hydrophilic interactions to IscS-TusA complex formation was demonstrated by disruption of the complex through increasing the NaCl concentration to 600 mM (unpublished data).

**Table 3:** Properties of IscS mutant proteins as assessed from pull-down and in vivo complementation experiments.

IscS Protein	TusA^a^	mnm^5^s^2^U (TusA)^b^	IscU^a^	s^2^C (IscU)^b^	ThiI^1^	s^4^U (ThiI)^b^	IscX^a^	CyaY^a^	Reference
WT		100		100		100			This work
R39A	nd	72		99		95			This work
R39E	Yes	nd	Yes		Yes	94	Yes	Low	This work
W45R	No	3, *4* c	Yes	96, *76*	Low	7, *0.5*	Yes	Yes	This work, [21]
E49A	No	24	Yes	93	nd	99	nd	nd	This work
D52A	No	11	Yes	104	nd	103	nd	nd	This work
D52R	No	0	Yes	102	nd	83	Yes	Yes	This work
D52Y	No	20	Yes	102	nd	102	nd	nd	This work
D52M	No	11	Yes	104	nd	103	nd	nd	This work
D65F	Low	*22*	Yes	*94*	Yes	*60*	Yes	Yes	This work, [21]
F89E	nd		Yes		Low		nd	nd	This work
R112E	nd		Yes		Yes		Low	nd	This work
R116E	Yes		Yes		Low		Low	Low	This work
R220E	Yes		Yes		No		No	No	This work
R223E	nd		nd		Low		No	No	This work
G234L	nd		Yes		Yes		Low	Low	This work
E311R	nd		Yes		Low		Yes	nd	This work
A327V	Yes	*48*	Low	*26*	Low	*26*	No	Low	This work, [21],[43]
C328S	nd		Yes		Yes		nd	nd	
R340E	Low		Yes		No		Low	No	This work
D346R	nd		Yes		Yes		Yes	nd	This work
L386R	nd		Yes		Yes		Yes	nd	This work
M389R	nd		Yes		nd		nd	nd	This work
R223E/R225E	nd		Yes		nd		No	nd	
R225E/E227R	Yes		Yes		Yes		No	No	This work
R237E/M239E	nd		Yes		No		No	No	This work
H96Y		*69*		*100*		*108*			[21]
M169V		*78*		*36*		*66*			[21]
A321S		*109*		*88*		*62*			[21]
S323A		*100*		*100*		*90*			[44]
S326A		*90*		*10*		*90*			[44]
L333A		*110*		*20*		*100*			[44]
S336A		*100*		*100*		*90*			[44]
H350R		*98*		*94*		*92*			[21]
Δ374-404			*Low*						[39]

a Interaction between the indicated His-IscS protein (prey) and wild-type GST-TusA, IscU, ThiI, IscX, and CyaA (baits). Yes, binding observed; Low, significantly less prey pulled down; No, no binding.

b Levels of s^2^C, mnm^5^s^2^U, and s^4^U in IC6087 transformed with pMJ623 derivative plasmids were determined in the absence of the IPTG inducer. Levels of the indicated thionucleosides were measured as the ratio of peak area to that of guanosine and expressed as a percentage of the wild-type values (IC6087/pMJ623). The numbers represent the mean values of at least three independent experiments. All nucleosides were quantified at 314 nm. Mutations R39A, W45R, E49A, D52A, D52R, D52Y, and D52M did not impair production of ms^2^i^6^A, which was quantified at 254 nm (unpublished data).

c Numbers in italics taken from the references shown in the last column.

Of the IscS mutations, only A327^IscS^V had some impact on IscU binding (Table 3 and Figure 7B). This mutation affects the residue next to Cys328, and the tip of this loop was disordered in our structure. No other IscS mutations investigated here affected IscS-IscU complex formation and the structure shows that all of these mutations are outside of the IscU interface with IscS (Figure 6B). However, an IscS(Δ374-404) deletion was reported to abrogate IscU binding [39], and this segment forms part of the interface observed in the structure. The agreement between the pull-down experiments and the crystallographically determined interfaces substantiated the results presented below for other proteins interacting with IscS.

*E. coli* ThiI is significantly larger than either TusA or IscU, with 482 residues arranged into three domains [29]. The ThiI residue Cys456 was shown to be essential for accepting sulfur from IscS [41],[42] and is located in the rhodanese-like domain. The mutants R220^IscS^E, R237^IscS^E/M239^IscS^E, and R340^IscS^E significantly decreased binding of ThiI, while the mutations W45^IscS^R, F89^IscS^E, R116^IscS^E, R223^IscS^E, E311^IscS^R, and A327^IscS^V decreased binding to a lesser extent (Figure 7C and Table 3). Therefore, binding of TusA or ThiI to IscS is influenced by a common mutation, W45^IscS^R, indicating that they bind to distinct but partially overlapping regions on the IscS surface.

The binding of frataxin/CyaY and IscX to IscS was affected by the same set of mutations, including R116^IscS^E, R220^IscS^E, R223^IscS^E, R225^IscS^E/E227^IscS^R, G234^IscS^L, R237^IscS^E/M239^IscS^E, A327^IscS^V, and R340^IscS^E (Figure 7D,E and Table 3), showing that their footprints are very similar. Moreover, their footprints overlap significantly with that of ThiI but not with that of IscU nor TusA.

The effect of IscS mutations on binding to partner proteins was analyzed in vivo by quantification of the tRNA modifications mnm^5^s^2^U (TusA), s^2^C (IscU), and s^4^U (ThiI). To this end, we used an *iscS* null mutant (IC6087) transformed with pMJ623 and derivative plasmids, which encode the wild-type and mutant His-IscS proteins, respectively. We decided to use this approach after observing that plasmid pMJ623 was able to restore the nearly wild-type levels (90%) of thiolated nucleosides when transformed into IC6087, despite that His-IscS could not be detected with anti-His antibody in Western blot analysis (unpublished data). Mutations W45^IscS^R, E49^IscS^A, D52^IscS^A, D52^IscS^R, D52^IscS^Y, and D52^IscS^M reduce the mnm^5^s^2^U synthesis to 0%–25% of the wild-type protein, whereas they do not affect s^2^C accumulation. These results correlate well with the effect produced by such mutations on the IscS interaction with TusA and IscU, as assessed by the pull-down experiments (Table 3), suggesting that the impairment or complete inability of IscS mutants to bind TusA is responsible for the decrease in mnm^5^s^2^U modification. The mutation A327^IscS^V does not interfere with the pull-down of IscS by TusA, although it reduces the mnm^5^s^2^U synthesis by about 50% [21],[43].

The mutation W45^IscS^R decreases both mnm^5^s^2^U and s^4^U levels to about 5% of the wild-type protein, confirming that Trp45 affects binding to TusA and ThiI (Table 3 and [21]). However, other mutations impairing the interaction with TusA (E49^IscS^A, D52^IscS^A, D52^IscS^Y, and D52^IscS^M) do not reduce synthesis of s^4^U, suggesting that they do not abrogate the interaction with ThiI. These results support that TusA and ThiI bind to distinct but partially overlapping regions on the IscS surface. Taken together with the determined structures, the in vitro and in vivo experiments enabled us to create a protein interaction map of the IscS surface (Figure 6B).

### IscS Can Bind Multiple Partners Simultaneously

Structures of the IscS-TusA and IscS-IscU complexes showed that the footprints of TusA and IscU on the IscS surface do not intersect. Therefore, we applied a three-way pull-down approach to explore whether both of these proteins could bind simultaneously to IscS. We first incubated His_6_-IscS with GST-TusA on glutathione Sepharose beads, washed the beads extensively, and eluted the His_6_-IscS-TusA complex by cleavage with TEV protease. We then bound GST-IscU on fresh glutathione Sepharose beads, washed, and added the His_6_-IscS-TusA complex. The column was washed, TEV protease added, and incubated for ∼2 h. Only His-IscS and IscU eluted from the column (Figure S6A, left). In the second experiment, we first formed the His_6_-IscS-IscU complex and loaded it on a glutathione Sepharose column pre-bound with GST-TusA. In the flowthrough we detected His-IscS-IscU. All of the GST-TusA and a small amount of His-IscS were retained on the beads (Figure S6A, right). In both experiments IscS associated predominantly with IscU, indicating that TusA and IscU cannot bind to IscS simultaneously and that IscU is able to displace TusA from IscS. The biological significance of this binding preference has to be investigated further. Subsequently, we performed three-way pull-down experiments for other protein-protein combinations with IscS, including IscU-CyaY (Figure S6B) [13],[14], IscU-IscX (Figure S6C), TusA-IscX (Figure S6D), and TusA-CyaY (Figure S6E). The results show that IscU can bind IscS simultaneously with either CyaY or IscX, whereas TusA cannot.

To determine if simultaneous binding of CyaY (or IscX) and IscU to IscS affects sulfur transfer to IscU, we examined the level of IscU-dependent s^2^C tRNA modification when CyaY (or IscX) was overexpressed for 18 h. No effects were found (unpublished data).

### Modeling the IscS-CyaY/IscX Complexes

**Figure 8:**
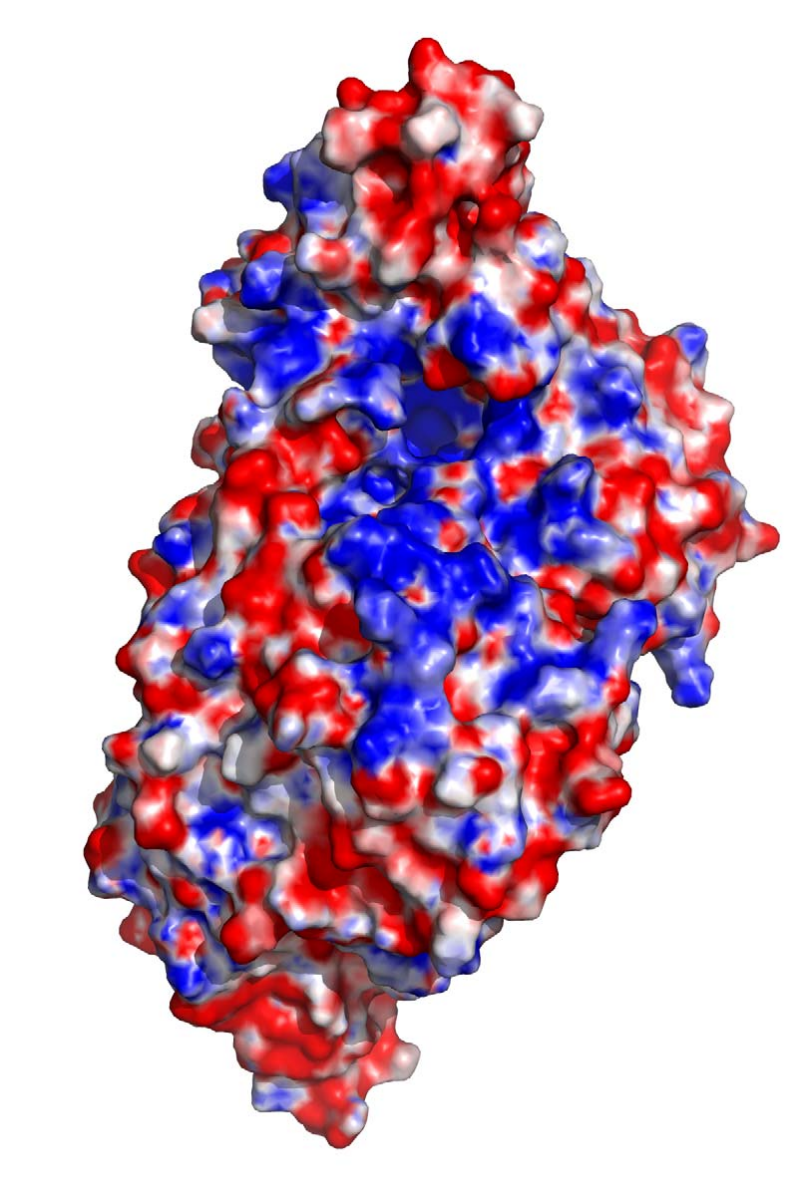
The electrostatic potential of the IscS dimer: red, negative; blue, positive. Surface with positive potential overlaps with the footprint of CyaY and IscX. Orientation similar to that in Figure 6B.

**Figure 9:**
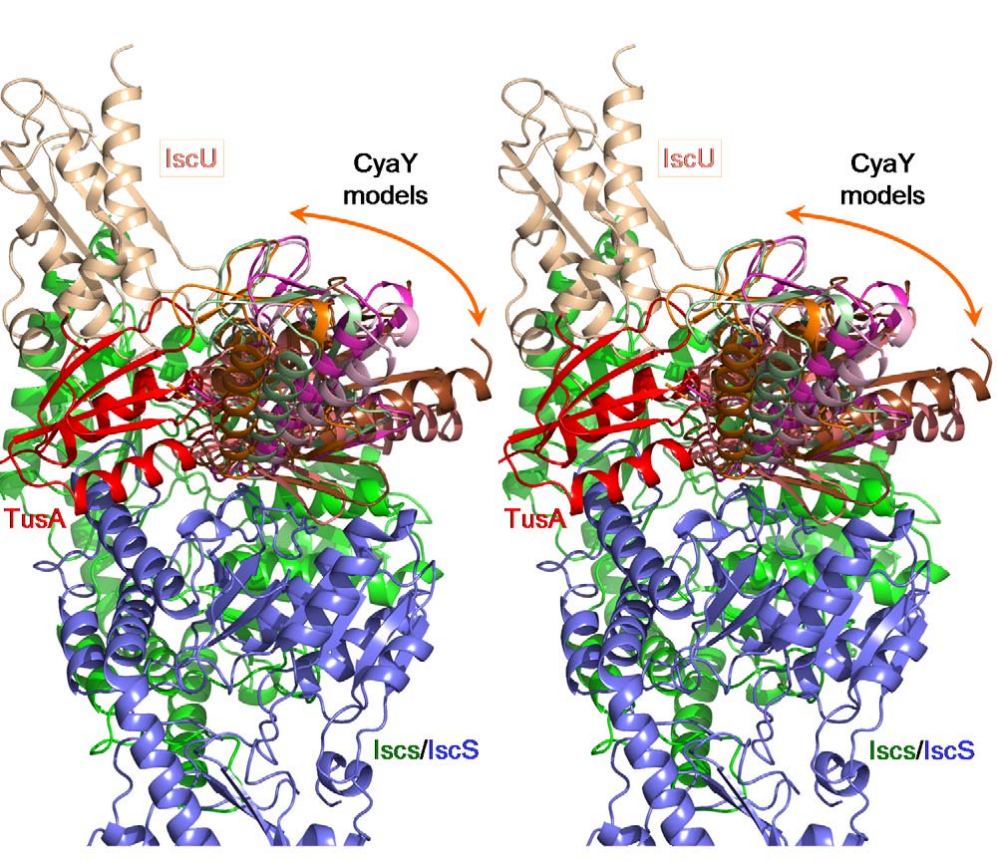
Modeling of the IscS-CyaY complex using the interface residues on CyaY identified by NMR [14] and residues of IscS important for binding to CyaY as identified here. The top 6 CyaY models are shown. The IscS subunits are painted green and slate. The overlapping CyaY models are shown in different colors. The locations of IscU (wheat) and TusA (red) relative to IscS are also shown. There are no steric conflicts between IscU and any of the CyaY models. The TusA molecule, however, clashes with all of the top models of CyaY, as was expected from the competition experiments.

As previously observed, both CyaY and IscX contain a large, negatively charged patch on their surface that has been proposed to contain residues involved in binding to IscS [14],[16],[37]. The CyaY and IscX footprints on the IscS surface encompass a positively charged area (Figure 8). We have used the ZDOCK server (http://zdock.bu.edu/) to model the IscS-CyaY and IscS-IscX complexes. In the first approach no restraints were provided. While the 20 top solutions positioned CyaY over the positively charged surface of IscS near the Cys328 loop, the orientation of CyaY varied significantly and all the top solutions collided with IscU. In the second approach we provided CyaY residues identified by NMR [14] as restraints. Again, more than half of the 20 best models collided with IscU. However, when we added IscS restraints derived from pull-down assays, none of the top 20 solutions clashed with IscU, and the range of CyaY orientations was smaller than in the previous calculations (Figure 9 and Figure S7). What is more, all of the CyaY models collided, albeit slightly, with the TusA structure (Figure 9). This is consistent with the detection of an IscS-IscU-CyaY ternary complex and the lack of detection of an IscS-TusA-CyaY complex. Similar modeling results were obtained for IscX (unpublished data).

### Querying the Roles of Conserved TusA Residues in Sulfur Transfer

The crystal structures presented here allow us to address the mechanism of sulfur transfer from IscS to acceptor proteins. In the IscS-TusA complex, the observed proximity of Cys19^TusA^ to persulfated Cys328^IscS^ could be sufficient for sulfur transfer to occur. However, several residues, including Asp45^TusA^ and Asp51^TusA^ in the vicinity of Cys19^TusA^, are absolutely conserved and could play a role in sulfur transfer (Figure 3A). Asp51^TusA^ is on the surface while Asp45^TusA^ is buried but forms a hydrogen bond to the NH of Cys19^TusA^. To investigate their roles, we constructed mutations D45^TusA^A and D51^TusA^A as well as other mutations affecting TusA residues in proximity to IscS, E21^TusA^A, M24^TusA^R, R27^TusA^E, R27^TusA^D, R31^TusA^A, and F58^TusA^A, and tested each mutant for IscS-TusA complex formation in vitro (Figure S5B) and in vivo for levels of TusA-dependent mnm^5^s^2^U tRNA modification (Table 4) [23].

**Table 4:** Properties of TusA mutant proteins as assessed from pull-down and in vivo complementation experiments.

GST-TusA Protein	IscS Interaction^a^	mnm^5^s^2^U^b^
WT	Yes	2.7±0.15 (100)
E21A	Low	Not tested
M24R	No	1.5±0.1** (56)
R27E	No	0.5±0.14*** (19)
R27D	No	Not tested
R27D/P35S	Not tested	0
R31A	No	0.8±0.15*** (30)
F58A	No	1.8±0.1** (67)
D45A	Yes	1.5±0.08** (56)
R50A	Yes	1.8±0.08** (67)
D51A	Yes	1.8±<0.01** (67)

a Interaction determined by pull-down between the indicated GST-TusA protein (prey) and His-IscS (bait). Yes, binding observed; No, no binding.

b Synthesis of mnm^5^s^2^U was analyzed in strain IC6085 (BW25113 *tusA::kan*) harbouring pMJ683 and derivatives, which express the wild-type and mutant GST-TusA versions, respectively, of GST-TusA. Levels of mnm^5^s^2^U were measured as the ratio of peak area to that of guanosine, quantified at 314 nm. Nucleoside mnm^5^s^2^U was undetectable in IC6085 and IC6085 carrying pGEX 4T-1, whereas its level reached a value of 3.1 in BW25113 (wild-type strain). Values are expressed as the mean ± standard error from at least three independent experiments. Statistical comparison among groups was carried out by the Student's test. Differences from the wild-type value were considered significant at *p<0.05, **p<0.005, and ***p<0.0005. Numbers in parentheses are the levels of the nucleoside expressed as a percentage of the wild-type value (IC6085/pMJ683).

For the in vivo experiments we followed the synthesis of mnm^5^s^2^U in a *tusA* null mutant (IC6085) transformed with pGEX 4T-1 (expressing only GST) and derivative plasmids expressing wild-type or mutant GST-TusA proteins. Western blot analysis with an anti-GST antibody indicated that the recombinant proteins are synthesized even in the absence of the IPTG inducer, due to leakiness of the P_tac_ promoter, and that the cellular levels of the GST-TusA protein produced by each recombinant plasmid under such conditions were similar (unpublished data), suggesting that the introduced mutations did not affect stability of the GST-TusA protein. In all cases where the mutants show weak or no interaction in the pull-down assay, the level of tRNA modification also decreases (Table 4). Even when we detected no interaction by in vitro pull-downs, the remaining low IscS-TusA affinity seems to be sufficient to provide partial complementation over the several hours of cell growth, accounting for the reduced levels of tRNA modification observed in such cases (Table 4).

The TusA interface mutations M24^TusA^R, R27^TusA^E, R27^TusA^D, R31^TusA^A, and F58^TusA^A abolished in vitro binding to IscS, while E21^TusA^A only weakened complex formation with IscS (Table 4). A more sensitive technique, surface plasmon resonance (SPR), did not detect interaction between His-IscS and several of these TusA mutants (M24R, R27E, R31A, F58A) (unpublished data). On the other hand, the D51^TusA^A and D45^TusA^A mutants behaved like wild-type TusA in the pull-down experiments with IscS, showing that these mutations had little or no effect on IscS-TusA complex formation (Table 4). When assayed in vivo, D51^TusA^A and D45^TusA^A showed reduced levels of mnm^5^s^2^U modification, to 67% and 56%, respectively, of that of the wild-type TusA (Table 4), supporting a functional role for Asp45 and Asp51.

## Discussion

### Molecular Footprints on the IscS Surface

IscS and several of its binding partners are evolutionarily highly conserved proteins. In order to characterize at the molecular level the mode of interaction of IscS with its binding partners and to define their footprints on the IscS surface, we determined the crystal structures of IscS with two sulfur acceptors, IscU and TusA. We also utilized data from the literature for 9 mutations [21],[39],[44] with over 20 mutations investigated here to map interactions for three other proteins, ThiI, CyaY/frataxin, and IscX. We identified multiple mutations that disrupted binding for each of the partners (Table 3). The in vivo effects largely coincide with the in vitro binding studies (Table 3), offering supporting evidence that disrupting the interactions of IscS with its partners impairs tRNA modification. The structures of the IscS-TusA and IscS-IscU complexes validated this methodology.

The footprints of ThiI, CyaY, and IscX overlap significantly, while ThiI and TusA overlap partially (Figure 6B). Our results indicate that CyaY and IscX bind to nearly the same region of IscS. Although the TusA and IscU footprints do not overlap, the three-way pull-down experiments showed that TusA and IscU cannot bind simultaneously to IscS. Moreover, IscU was able to displace TusA in the complex, suggesting that it has a higher affinity for IscS. Superposition of the structures of these two IscS complexes shows, indeed, a spatial overlap between bound IscU and TusA (Figure S4). Taken together, our data show that the sulfur acceptors IscU and TusA and ThiI can bind to IscS only one at a time and that the effectors/modulators CyaY/frataxin and IscX can form a ternary complex with IscS in the presence of IscU but not with TusA or ThiI.

As CyaY and IscU can both bind to IscS simultaneously, we asked if CyaY may prevent IscU from acquiring sulfur from IscS in vivo. To determine this we overexpressed CyaY or IscX in a wild-type *E. coli* strain and quantified the level of the modified s^2^C nucleotide, finding that overexpression has no effect on s^2^C synthesis under our growth conditions (unpublished data).

Several, and often contradictory, views on the role of frataxins have been proposed. Thus, Frataxin/CyaY has been postulated as an Fe chaperone [18], an Fe donor for Fe-S cluster assembly [13],[19],[20], or a regulator of Fe-S cluster formation [14]. Since we did not detect impairment in s^2^C modification under CyaY overproducing conditions, it may be concluded that CyaY does not interfere with sulfur transfer between IscS and IscU under standard growth conditions, which favours the view of CyaY as a source of Fe via IscU for Fe-S cluster assembly. However, some biochemical studies on frataxins suggest that their activity might be modulated in vivo by the intracellular iron concentration [14] or redox potential [19]. Therefore, additional experiments are needed to test the effect of the CyaY overexpression under such conditions.

### Mode of Interaction of the IscS Dimer with the Acceptor Proteins: IscU Acts in Cis While TusA Acts in Trans

Each IscU molecule interacts with only one subunit of the IscS dimer and, based on its orientation in the complex, would be expected to accept sulfur from the same subunit to which it is bound (Figure 2). Of the three cysteines in IscU, the closest to the loop bearing Cys328^IscS^ is Cys37^IscU^. The tip of the IscS loop is disordered and we cannot precisely position Cys328^IscS^, however the distance of ∼12 Å estimated from the model would be too far for sulfur transfer. The other two cysteines are slightly further away, with distances of ∼13.5 Å for Cys63^IscU^ and ∼16 Å for Cys106^IscU^. Therefore, an additional movement, most likely of the IscS loop, is required to bring the catalytic Cys residues closer together.

The mode of TusA interaction with IscS is different. While TusA interacts predominantly with one IscS subunit, the sulfur accepting Cys19^TusA^ [23] is juxtaposed against Cys328′^IscS^ that belongs to the other IscS subunit of the dimer (Figures 2, 3A). As a result, the thiol groups of Cys328′^IscS^ and Cys19^TusA^ are in close proximity, within a distance of less than 4.5 Å. This organization of the IscS-TusA complex suggests that the dimerization of IscS is essential for effecting sulfur transfer to various acceptor proteins.

### The High Flexibility of the Cys328^IscS^ Loop Is Crucial for Sulfur Transfer to Multiple Acceptors

While the catalytic mechanism of cysteine and selenocysteine desulfurase/deselenase activity has been intensively investigated [45]–[48], less is known about how persulfide sulfur is transferred to an acceptor protein. Evidence suggests that the cysteine persulfide intermediate is a relatively stable species and represents a true enzyme intermediate along the reaction pathway [1],[49].

The loop containing Cys328^IscS^, which would carry the persulfide, extends away from the PLP cofactor and the cysteine-binding site, but the location of its tip harbouring Cys328^IscS^ could not be detected due to disorder [27]. We have determined the structure of PLP-bound IscS at 2.05 Å resolution in a different crystal environment from that observed previously and have also found the Cys328^IscS^-containing loop extending away from the protein with its tip disordered. Therefore, IscS prefers an “open” conformation of the Cys328 loop, compatible with sulfur transfer to an acceptor. In contrast, the analogous loops in two other cysteine desulfurases, NifS and SufS, are shorter and prefer a closed conformation, with the active site cysteine residue located in proximity to PLP, compatible with loading of sulfur acquired from bound cysteine substrate (Figure S8). We postulate that the longer Cys328 loop found in IscS is essential for this enzyme to transfer sulfur to multiple acceptors.

We propose that the transfer of persulfide sulfur from IscS to the acceptor occurs in two stages. In the first stage, the loop containing Cys328 assumes the “closed” conformation and is loaded with the sulfur acquired from the cysteine substrate via the PLP cofactor, as exemplified by the structure of SufS/CsdB [49]. Next, the Cys328-carrying loop pivots around hinges located near Ser324 and Ser336, adopting the “open” conformation such that Cys328 can closely approach the cysteine of the acceptor protein. The conformation of the Cys328^IscS^ loop in the IscS-TusA complex, with the donor and acceptor cysteines in close proximity, suggests that the observed conformation is close to that expected in a transfer-competent state (Figure 3A). This transfer mechanism is likely common with both NifS and SufS desulfurases.

IscS transfers sulfur to multiple acceptor proteins. In the complex with IscU the observed distance between Cys328^IscS^ and the Cys residues of IscU is too long for a direct transfer (Figure S4), and consequently a conformational rearrangement is necessary to bring together the sulfur donor and acceptor cysteines. Since most regions of IscS show no differences in the various crystal structures, either alone or complexed with acceptor proteins, and in view of the high flexibility/disorder of the Cys328 loop, we postulate that it is this loop that bends closer toward IscU in order to effect sulfur transfer. Indeed, the observation of a disulfide linkage between Cys328^IscS^ and Cys37^IscU^ from *Azotobacter vivendi* [50] or with *E. coli* Cys63^IscU^ [51] supports the notion that the Cys328 loop travels over a significant distance in order to interact with different partners. This implies that the flexibility of the Cys328 loop is crucial for the IscS ability to act as a shuttle in sulfur transfer and is consistent with the in vivo effects of mutations in the loop region of IscS on Fe-S cluster synthesis [21],[43],[44]. Our observation that the A327^IscS^V mutation weakens the IscS interaction with IscU, ThiI, CyaY, and IscX is also compatible with this hypothesis. Given that Ala327^IscS^ is adjacent to the catalytic Cys328^IscS^, the mutation A327^IscS^V likely affects the flexibility of the active loop, resulting in impaired binding of IscS to some of its partners.

The modeled position of Cys328^IscS^ is closer to Cys37^IscU^ and Cys63^IscU^ than to Cys106^IscU^. The sidechain of Cys37^IscU^ is exposed on the protein surface, with Cys63^IscU^ being less exposed while Cys106^IscU^ is buried. We propose that the most likely candidate residue to act as the initial S acceptor is Cys37^IscU^ followed by Cys63^IscU^. The distance between the sidechains of Cys63^IscU^ and Cys106^IscU^ is ∼4 Å, allowing for a secondary transfer of persulfide sulfur from Cys63^IscU^ to Cys106^IscU^. The observation that mutation of any one of the IscU cysteines reduced the number of sulfurs bound to IscU but did not abolish sulfur transfer [50] indicates that more than one cysteine can accept the sulfur directly from IscS.

### The Role of Additional Residues in Assisting Sulfur Transfer

We questioned if sulfur transfer between the two cysteines requires assistance from other residues. We noted that Asp45^TusA^ and Asp51^TusA^ are close to Cys19^TusA^ (Figure 3A) and are conserved in all homologs with sequence identity > ∼24%. The sidechain of Asp45^TusA^ forms a hydrogen bond to the NH of Cys19^TusA^ that may be helpful to correctly orient the loop carrying this cysteine. The sidechain of Asp51^TusA^ is 4.2 Å away from the sulfur of Cys19^TusA^. The expected chemistry requires that Cys19^TusA^ acts as a nucleophile attacking the Cys328^IscS^ persulfide, for which Cys19^TusA^ would be more reactive if it were deprotonated [1]. While at neutral pH a small fraction of cysteines would be deprotonated, we rationalized that Asp51^TusA^ could act as a general base to deprotonate Cys19^TusA^. The D51^TusA^A mutation modestly affects sulfur transfer, as measured by the level of mnm^5^s^2^U modification in vivo, whereas, as expected, it does not impede IscS-TusA complex formation in vitro (Table 4). Therefore, we postulate that while Asp51^TusA^ is not absolutely essential, it makes Cys19^TusA^ more nucleophilic, increasing the enzyme's efficiency and resistance to changes in pH. The sulfuryl anion would also be stabilized by the nearby Arg50^TusA^. This residue, while only moderately conserved, also has a functional role as the R50^TusA^A mutant shows reduced tRNA modification without affecting IscS-TusA complex formation (Table 4). While our proposal is in agreement with the current data, more detailed investigations of the sulfur transfer reaction in vitro will be needed to establish the roles of the above-mentioned residues.

Interestingly, an aspartate (Asp39^IscU^) has also been shown to destabilize the Fe-S cluster in IscU [31],[38]. Mutation of this aspartate to an alanine was essential for crystallization of the *Aquifex aeolicus* IscU-(Fe-S)_2_ cluster. This aspartate is located in between Cys37^IscU^, Cys63^IscU^, and Cys106^IscU^ and we hypothesize that, by analogy to Asp51^TusA^, it could also participate in catalysis.

### Functional Implications

Our combined biochemical and structural studies provide the first molecular details of how IscS both recognizes and discriminates between various binding partners. IscS binds its partners via a large, highly conserved, contiguous docking surface extending across both IscS subunits and centered on the loop containing Cys328. Different binding partners utilize different parts of this docking surface and approach Cys328 from different directions. The key to the ability of IscS to transfer persulfide sulfur to multiple acceptor proteins is the length and flexibility of the loop carrying Cys328. Indeed, superposition of the complexes shows that Cys19^TusA^ and Cys37^IscU^ are over 16 Å apart (Figure S4), yet both can accept sulfur from Cys328^IscS^. The shorter loops carrying the active site cysteine in SufS and NifS are likely adapted for interaction with only a single acceptor protein, SufU and NifU, respectively, and may require the binding of this partner to trigger flipping of this loop from an inside conformation to an outside one.

It is clear that IscS binds the monomeric form of apo-IscU, consistent with the model proposed by Shimomura et al. [31], and would be structurally inconsistent with binding of an IscU trimer containing an Fe-S cluster. It is also noteworthy that the binding site on IscU for the HscA chaperone, required for Fe-S cluster assembly or delivery from IscU to target proteins, may have some overlap with that for IscS since Lys103^IscU^ was shown to be involved in HscA binding [52] and the K103^IscU^E mutation also disrupts the IscS-IscU complex (Figure S5A and Table 2). This argues against simultaneous binding of IscU to IscS and HscA and is consistent with a role for this chaperone in mediating delivery of the Fe-S cluster to recipient proteins. On the other hand, the IscU binding site for the co-chaperone HscB [53] is distinct from that for IscS and HscB could interact with the IscU-IscS complex. Since formation of an Fe-S cluster likely occurs while IscU is bound to IscS [38] and HscA affinity for IscU increases ∼20-fold in the presence of HscB [54], a plausible model is that HscB promotes dissociation of the IscS-IscU(Fe-S) complex and a formation of an IscU(Fe-S)-HscB-HscA complex for subsequent transfer of the Fe-S cluster to a recipient protein.

Within the cell, the relative affinities of partner proteins for the IscS dimer, their Fe-loading state (IscU, CyaY, and IscX), as well as their relative concentrations together presumably dictate which combination(s) of partner proteins interact with IscS at any one time. The simultaneous binding of TusA and IscU to IscS, while it involves different surface residues on IscS, is precluded due to steric clashes. The higher affinity of IscS for IscU than for TusA suggested by our results is of functional importance in that under conditions of limited sulfur supply, sulfur would be delivered predominantly to IscU, the precursor for Fe-S cluster assembly. The overlapping footprints of ThiI and TusA on the IscS surface suggests that they cannot bind IscS simultaneously and, therefore, implies that synthesis of modified tRNAs containing S^4^U and S^2^U depends on binding competition between these two proteins. The pertinent question of the precise order of events at the molecular level leading to Fe-S cluster assembly on IscU, with respect to donation of Fe and S atoms, remains an area for further research.

## Materials and Methods

### Cloning, Expression, and Purification

The *iscS* gene (NCBI gi: 12516934) from *E. coli* O157:H7 EDL933 [55] was cloned into a modified pET15b vector (Novagen) and was expressed in *E. coli* BL21(DE3), yielding a fusion protein with an N-terminal His_6_-tag. The *tusA* (NCBI gi:12518129), *iscU* (gi:12516933), *thiI* (gi:26106827), *iscX* (gi:12516925), and *cyaY* (gi:12518674) genes from the same bacterium were cloned into a modified pGEX-4T1 vector (GE Healthcare, Baie d'Urfe, Quebec, Canada) and expressed in *E. coli* BL21 as N-terminal glutathione *S*-transferase (GST) fusion proteins with a tobacco etch virus (TEV) protease cleavage site for removal of the tag. For each protein, an overnight culture of transformed *E. coli* BL21 was used to inoculate a 11 culture in TB medium containing 100 µg/ml ampicillin. The culture was grown at 37°C until the absorbance at 600 nm reached 0.6. Protein expression was induced with 100 µM isopropyl 1-thio-β-D-galactopyranoside (IPTG) followed by incubation for 16–20 h at 20°C. Cells were harvested by centrifugation (4,000×g, 4°C, 25 min) and stored at −20°C. The cell pellet was re-suspended in 40 ml of lysis buffer (50 mM Tris-HCl pH 8.0, 0.15 M NaCl, 5% (v/v) glycerol). To obtain the IscS-TusA complex, the cell pellets of His_6_-IscS and GST-TusA were mixed and disrupted by sonication (12×10 s, with 10 s between bursts). Cell debris was removed by centrifugation (33,000×g, 45 min, 4°C). The protein supernatant was loaded onto a 2 ml bed volume of glutathione Sepharose resin (GE Healthcare, Mississauga, Canada) equilibrated with lysis buffer. Beads were washed with 4 column volumes of TEV cleavage buffer (50 mM Tris pH 8.0, 150 mM NaCl, 0.5 mM EDTA) to remove unbound proteins and excess IscS protein. The complex was released from the column by cleavage with TEV protease (1∶100 [wt/wt]) for 3 h at room temperature. The IscS-TusA complex was further purified by size exclusion chromatography (SEC) on a Hi-Load Superdex 200 16/60 column (GE Healthcare) equilibrated in a buffer containing 20 mM Tris-HCl pH 8, 150 mM NaCl, 2% (v/v) glycerol. Fractions containing the protein complex were pooled and concentrated to 35 mg/ml. The IscS-IscU complex was purified in a similar manner. Dynamic light scattering measurements were performed at room temperature using a DynaPro plate reader (Wyatt Technologies, Santa Barbara, CA).

### Crystallization

Initial crystallization conditions were found by sitting drop vapour diffusion at 21°C using Qiagen JCSG Core Suite screens (Qiagen, Mississauga, Canada) and optimized by hanging drop vapour diffusion methods. The best crystals of IscS-TusA were grown by equilibrating 1 µl of protein (35 mg/ml) in buffer (20 mM Tris-HCl pH 8, 150 mM NaCl, 2% (v/v) glycerol) mixed with 1 µl of reservoir solution (0.12 M magnesium formate, 20% [w/v] PEG 3350) suspended over 1 ml of reservoir solution. Two crystal forms were obtained under the same crystallization conditions. Crystals for form 1 are orthorhombic, space group *P*2_1_2_1_2_1_, with *a* = 72.3, *b* = 106.5, *c* = 122.1 Å, with an IscS dimer and two TusA molecules in the asymmetric unit and V_m_ = 2.05 Å^3^ Da^–1^ [56]. Crystals of form 2 are also orthorhombic, space group *C*222_1_, with *a* = 72.9, *b* = 131.4, *c* = 106.4 Å, with one IscS subunit and one TusA molecule in the asymmetric unit and V_m_ = 2.28 Å^3^ Da^–1^. Crystals of IscS-PLP were obtained from 0.1 M Bicine pH 8.5, 15% (w/v) PEG 6000 and belong to space group *P*2_1_2_1_2_1_, with *a* = 74.8, *b* = 99.2, *c* = 118.1 Å, and V_m_ = 2.43 Å^3^ Da^–1^.

The best crystals of the IscS-IscU complex were obtained by hanging drop vapour diffusion by mixing 1 µl of IscS-IscU (30 mg/ml) in buffer (20 mM Tris-HCl pH 8, 100 mM NaCl, 2% v/v glycerol) with 1 µl of reservoir solution (0.2 M sodium nitrate, 16% [w/v] PEG 8000, 4% [v/v] glycerol, 0.1 M Bicine pH 9) and equilibrated over reservoir solution. The complex crystallizes in space group *P*6_1_22, with unit cell dimensions *a,b* = 77.6 Å, *c* = 356.0 Å, V_m_ = 2.59 Å^3^ Da^–1^, and one molecule of IscS and one molecule of IscU in the asymmetric unit.

### X-ray Data Collection, Structure Solution, and Refinement

For data collection, crystals were transferred to reservoir solution supplemented with 15% (v/v) ethylene glycol and flash cooled in a nitrogen stream at 100 K (Oxford Cryosystems, Oxford, UK). Diffraction data for both crystal forms of IscS-TusA were collected at the sector 31-ID beamline (LRL-CAT), Advanced Photon Source, Argonne National Laboratory. Data for the IscS-IscU crystal were collected at the CMCF 08ID beamline, Canadian Light Source, Saskatoon, Saskatchewan. Data integration and scaling were performed with HKL2000 [57]. The structures were solved by molecular replacement with the program Phaser [58] using the previously-reported *E. coli* IscS (PDB code 1P3W) and TusA (PDB code 1DCJ) structures as the search models. Refinement was carried out with the programs Refmac5 [59] and Phenix [60], and the models were improved by interspersed cycles of fitting with Coot [61]. The structures were refined applying group B-factors (one per chain for low resolution and one per residue for medium resolution). The translation-libration-screw (TLS) model was applied near the end of refinement. For IscS-TusA form 1 the final R-work is 0.222 and R-free is 0.240 at 2.45 Å resolution. The residues 327–332 and 391–404 in IscS subunit A, 329–332 and 393–404 in subunit B, and residues 1–3 and residue 81 in both TusA molecules are disordered and were not modeled. For crystal form 2 the R-work is 0.207 and R-free is 0.249 at 2.45 Å resolution. The residues 329–332 and 393–404 in IscS and 1–3 and 80–81 of TusA are disordered and were not modeled. The IscS-PLP structure was refined at 2.05 Å resolution to R-work of 0.198 and R-free of 0.239. The disordered region included residues 328–332 and 399–404 in chain A and 328–332 and 394–404 in chain B. In all IscS molecules the loop 322–333, carrying the essential catalytic Cys328 that accepts the S atom in the persulfated form, extends away from the body of IscS and is less well ordered. The structure of the IscS-IscU complex was also solved by molecular replacement with the same search model for IscS and using the IscU search model (PDB code 2Z7E) with program Phaser and was refined using tight geometric restraints at 3.0 Å resolution to R-work of 0.225 and R-free of 0.269. The residues 328–332 and 394–404 in IscS and residues 1, 127–128 in IscU were not modeled. In each structure the tips of several sidechains, mostly lysines, arginines, and glutamates, were also disordered and were not included in the models. All models have good stereochemistry (Table 1) as analyzed with PROCHECK [62].

Coordinates have been deposited in the RCSB Protein Data Bank with accession codes 3LVJ for IscS-TusA form 1, 3LVK for IscS-TusA form 2, 3LVL for IscS-IscU, and 3LVM for IscS structures, respectively. Data collection and refinement statistics are summarized in Table 1.

### SAXS Analysis

The SAXS measurements were carried out using an Anton Paar SAXSess camera equipped with a PANalytical PW3830 X-ray generator and a Princeton CCD detector. The beam length was set to 18 mm and the beam profile was recorded using an image plate for subsequent desmearing. Data for the IscS-IscU complex were collected at 4°C with protein concentrations of 4.5 mg/ml (10 h), 10 mg/ml (2 h), and 21 mg/ml (2 h). For the IscS-TusA complex, a data set was recorded at 4°C for 30 min at 22 mg/ml. Dark current correction, scaling, buffer subtraction, and desmearing were performed using the Anton Paar software SAXSquant 3.0. Data sets recorded at different concentrations for IscS-IscU were merged in Primus after removal of the lowest resolution shell (0.012–0.12 Å^−1^) for the 10 and 21 mg/ml data sets, for which Guinier plots showed larger R_g_ values (∼39 Å) indicating concentration-dependent oligomerization. The data sets were binned (5∶1) in the range of 0.012–0.35 Å^−1^ and fitted directly against predicted scattering calculated from atomic coordinates using the program CRYSOL (http://www.embl-hamburg.de/ExternalInfo/Research/Sax/crysol.html). Experimental R_g_ values were estimated from Guinier plots, while calculated R_g_ values were determined using CRYSOL.

### Mutagenesis of IscS, IscU, and TusA

Oligonucleotide primers were designed according to the QuikChange site-directed mutagenesis method (Stratagene) and synthesized by Integrated DNA Technologies. Using the plasmids carrying the wild-type genes as templates, the mutagenesis was performed according to the manufacture's instructions. *E. coli* DH5α was transformed with the mutagenized plasmids. Plasmids were isolated from the transformants and verified by DNA sequencing. *E. coli* BL21(DE3) were then transformed with plasmids containing the confirmed point mutations for protein expression.

### Pull-Down Studies of IscS with Binding Partners

Mutants of IscS and all binding partners were expressed following the same protocol used for the wild-type counterparts. To follow the interactions between IscS and its partners, we used His_6_-IscS and partner proteins fused to an N-terminal, TEV-cleavable GST tag. For a specific protein pair, cell pellets from 250 ml individual cultures were mixed, sonicated, centrifuged, and the protein supernatant loaded onto a 250 µl glutathione Sepharose column. Beads were washed with 3 column volumes of buffer (50 mM Tris-HCl pH 8, 200 mM NaCl, 2% (v/v) glycerol, except for CyaY where 50 mM NaCl was used). For the IscS-IscU pair, the GST-tag on IscU was cleaved prior to elution in order to distinguish its molecular weight from that of IscS. As a positive control, in each case co-purification of the wild-type protein complex was performed in parallel. Proteins retained on the beads or in the case of IscS-IscU, the eluted protein sample, were analyzed by SDS-PAGE.

### In Vivo Analysis of IscS and TusA Mutants

The *tusA* and *iscS* genes were deleted by targeted homologous recombination [63] using the oligonucleotide primers TusA(F), TusA(R), IscS(F), and IscS(R) (Table S1). The BW25113 [63] derivative strains were named IC6085 (BW25113 *tusA::kan*) and IC6087 (BW25113 *iscS::kan*). tRNA from the wild-type and mutant strains carrying pMJ623, pMJ683, or their derivative plasmids was purified and degraded to nucleosides as previously described [64]. The hydrolysate was analyzed by HPLC [65] using a Develosil C30 column (250×4.6 mm; Phenomenex Ltd). Western blot analysis to detect GST-TusA, GST-CyaY, GST-IscX, and GroEL proteins was performed with anti-GST (a generous gift from R. Pulido) and anti-GroEL antibodies (Calbiochem).

## Supporting Information

Figure S1 **Sequences with secondary structure assignments above: h, α-helix, s, β-strand.** Secondary structures are numbered in the middle of strings of sssss or hhhhh.
(0.58 MB TIF)

Figure S2 **Electron density for the IscS-TusA and IscS-IscU binding interface.** (A) Stereoview of the IscS-TusA interface with 2mF_o_-DF_c_ electron density shown at 1.0 σ level. The orientation is the same as in Figure 3A. The electron density for chain A of IscS is colored in blue, TusA in magenta. The electron density for the Cys328 loop in chain B of IscS is shown at 0.7 σ level and colored in green. For clarity the residues are not labelled. (B) Stereoview of the IscS-IscU interface with 2mF_o_-DF_c_ electron density shown at 0.9 σ level. The orientation is the same as in Figure 3B. The density for IscS is colored in blue, IscU in magenta. For clarity the residues are not labelled. This and other structural figures were prepared with the program PyMol (www.pymol.org).
(4.83 MB TIF)

Figure S3 **Conserved surface residues on IscU.** Top, cartoon representation; bottom, molecular surface. The level of conservation of surface residues is marked in shades of burgundy (dark, high conservation; white, highly variable).
(5.41 MB TIF)

Figure S4 **Superposition of Iscs-TusA and IscS-IscU showing the steric overlap of TusA and IscU.** The IscS subunits are painted green and cyan, TusA is magenta, and IscU is wheat. The IscS Cys328 loop is colored red and the cysteines in all molecules are shown explicitly in stick mode. The steric clashes would occur in the circled region. The separation between positions of acceptor cysteines of TusA and IscU is in excess of 16 Å and is marked with an arrow. The distance between the cysteines of IscS and TusA is ∼4 Å, while the distance between the cysteines of IscS and IscU is greater than 12 Å.
(3.20 MB TIF)

Figure S5 **Pull-downs of His-IscS by (A) IscU mutants and (B) TusA mutants.**
(2.35 MB TIF)

Figure S6 **Three-way pull-downs.** (A) Competition between TusA and IscU for IscS. Left: The preformed complex of His-IscS/GST-TusA was loaded on Glutathione Sepaharose 4B column (lane 1) and washed thoroughly. Beads incubated with TEV protease and released His-IscS-TusA complex eluted (lane 2). GST-IscU (lane 3) loaded on Glutathione Sepaharose 4B column and washed (lane 4), His-IscS-TusA added and column washed. Incubation with TEV protease and elution of released proteins (lane 5). Only His-IscS and IscU were observed. Right: Similar experiment performed in opposite order. First the His-IscS-IscU complex was formed and was loaded on the column with bound GST-TusA. His-IscS-IscU did not bind to the column and only GST-TusA was found on the beads. (B) Competition between IscU and CyaY for IscS. The preformed complex of His-IscS/GST-IscU was loaded on Glutathione Sepharose 4B column and washed thoroughly (lane 1). Since both proteins run at the same place on the SDS-PAGE, the beads were incubated with TEV protease and released proteins were eluted from the column (lane 2) confirming the presence of His-IscS-IscU complex. GST-CyaY was loaded on Glutathione Sepharose 4B column (lane 3) and His-IscS-IscU added to the column and washed (lane 4). Finally, incubation with TEV protease released His-IscS, IscU, and CyaY, showing that they formed a ternary complex. (C) Formation of a ternary complex between His-IscS, IscU, and IscX was determined using similar procedure as above. (D) Competition between TusA and IscX for IscS. Only His-IscS and IscX eluted after second TEV cleavage. (E) Competition between TusA and CyaY for IscS. Only His-IscS and CyaY eluted after second TEV cleavage.
(2.50 MB TIF)

Figure S7 **Models of the IscS-CyaY complex with restraints from NMR (CyaY) and mutagenesis (IscS).** The location of IscU (wheat) relative to IscS is also shown.
(4.96 MB TIF)

Figure S8 **Superposition of IscS and SufS.** The conformation of the loop bearing the active site cysteine is significantly different in both proteins.
(5.48 MB TIF)

Table S1 **Oligonucleotides used for construction of *iscS::kan* (IC6087) and *tusA::kan* (IC6085) strains.**
(0.04 MB DOC)

## Abbreviations

GST: glutathione *S*-transferase
IPTG: isopropyl 1-thio-β-D-galactopyranoside
*isc*: iron-sulfur clusters
*nif*: nitrogen fixation
PLP: pyridoxal-5′-phosphate
SAXS: small angle X-ray scattering
SEC: size exclusion chromatography
SPR: surface plasmon resonance
*suf*: sulfur mobilization
TEV: tobacco etch virus
TLS: translation-libration-screw

## References

[1] Mueller E. G (2006). Trafficking in persulfides: delivering sulfur in biosynthetic pathways.. Nat Chem Biol.

[2] Kessler D (2006). Enzymatic activation of sulfur for incorporation into biomolecules in prokaryotes.. FEMS Microbiol Rev.

[3] Johnson D. C, Dean D. R, Smith A. D, Johnson M. K (2005). Structure, function, and formation of biological iron-sulfur clusters.. Annu Rev Biochem.

[4] Ayala-Castro C, Saini A, Outten F. W (2008). Fe-S cluster assembly pathways in bacteria.. Microbiol Mol Biol Rev.

[5] Fontecave M, Ollagnier-de-Choudens S (2008). Iron-sulfur cluster biosynthesis in bacteria: mechanisms of cluster assembly and transfer.. Arch Biochem Biophys.

[6] Hu Y, Fay A. W, Lee C. C, Yoshizawa J, Ribbe M. W (2008). Assembly of nitrogenase MoFe protein.. Biochemistry.

[7] Zheng L, Cash V. L, Flint D. H, Dean D. R (1998). Assembly of iron-sulfur clusters. Identification of an iscSUA-hscBA-fdx gene cluster from Azotobacter vinelandii.. J Biol Chem.

[8] Zheng L, White R. H, Cash V. L, Jack R. F, Dean D. R (1993). Cysteine desulfurase activity indicates a role for NIFS in metallocluster biosynthesis.. Proc Natl Acad Sci U S A.

[9] Mihara H, Maeda M, Fujii T, Kurihara T, Hata Y (1999). A nifS-like gene, csdB, encodes an Escherichia coli counterpart of mammalian selenocysteine lyase. Gene cloning, purification, characterization and preliminary x-ray crystallographic studies.. J Biol Chem.

[10] Kambampati R, Lauhon C. T (1999). IscS is a sulfurtransferase for the in vitro biosynthesis of 4-thiouridine in Escherichia coli tRNA.. Biochemistry.

[11] Zhang W, Urban A, Mihara H, Leimkuehler S, Kurihara T (2010). IscS functions as a primary sulfur-donating enzyme by interacting specifically with MoeB and MoaD in the biosynthesis of molybdopterin in Escherichia coli.. J Biol Chem.

[12] Agar J. N, Krebs C, Frazzon J, Huynh B. H, Dean D. R (2000). IscU as a scaffold for iron-sulfur cluster biosynthesis: sequential assembly of [2Fe-2S] and [4Fe-4S] clusters in IscU.. Biochemistry.

[13] Layer G, Ollagnier-de C. S, Sanakis Y, Fontecave M (2006). Iron-sulfur cluster biosynthesis: characterization of Escherichia coli CYaY as an iron donor for the assembly of [2Fe-2S] clusters in the scaffold IscU.. J Biol Chem.

[14] Adinolfi S, Iannuzzi C, Prischi F, Pastore C, Iametti S (2009). Bacterial frataxin CyaY is the gatekeeper of iron-sulfur cluster formation catalyzed by IscS.. Nat Struct Mol Biol.

[15] Tokumoto U, Nomura S, Minami Y, Mihara H, Kato S (2002). Network of protein-protein interactions among iron-sulfur cluster assembly proteins in Escherichia coli.. J Biochem.

[16] Pastore C, Adinolfi S, Huynen M. A, Rybin V, Martin S (2006). YfhJ, a molecular adaptor in iron-sulfur cluster formation or a frataxin-like protein?. Structure.

[17] Forlani F, Cereda A, Freuer A, Nimtz M, Leimkuhler S (2005). The cysteine-desulfurase IscS promotes the production of the rhodanese RhdA in the persulfurated form.. FEBS Lett.

[18] Bou-Abdallah F, Adinolfi S, Pastore A, Laue T. M, Dennis C. N (2004). Iron binding and oxidation kinetics in frataxin CyaY of Escherichia coli.. J Mol Biol.

[19] Ding H, Yang J, Coleman L. C, Yeung S (2007). Distinct iron binding property of two putative iron donors for the iron-sulfur cluster assembly: IscA and the bacterial frataxin ortholog CyaY under physiological and oxidative stress conditions.. J Biol Chem.

[20] Li H, Gakh O, Smith D. Y, Isaya G (2009). Oligomeric yeast frataxin drives assembly of core machinery for mitochondrial iron-sulfur cluster synthesis.. J Biol Chem.

[21] Lundgren H. K, Bjork G. R (2006). Structural alterations of the cysteine desulfurase IscS of Salmonella enterica serovar Typhimurium reveal substrate specificity of IscS in tRNA thiolation.. J Bacteriol.

[22] Leipuviene R, Qian Q, Bjork G. R (2004). Formation of thiolated nucleosides present in tRNA from Salmonella enterica serovar Typhimurium occurs in two principally distinct pathways.. J Bacteriol.

[23] Ikeuchi Y, Shigi N, Kato J, Nishimura A, Suzuki T (2006). Mechanistic insights into sulfur relay by multiple sulfur mediators involved in thiouridine biosynthesis at tRNA wobble positions.. Mol Cell.

[24] Palenchar P. M, Buck C. J, Cheng H, Larson T. J, Mueller E. G (2000). Evidence that ThiI, an enzyme shared between thiamin and 4-thiouridine biosynthesis, may be a sulfurtransferase that proceeds through a persulfide intermediate.. J Biol Chem.

[25] Jager G, Leipuviene R, Pollard M. G, Qian Q, Bjork G. R (2004). The conserved Cys-X1-X2-Cys motif present in the TtcA protein is required for the thiolation of cytidine in position 32 of tRNA from Salmonella enterica serovar Typhimurium.. J Bacteriol.

[26] Pierrel F, Bjork G. R, Fontecave M, Atta M (2002). Enzymatic modification of tRNAs: MiaB is an iron-sulfur protein.. J Biol Chem.

[27] Cupp-Vickery J. R, Urbina H, Vickery L. E (2003). Crystal structure of IscS, a cysteine desulfurase from Escherichia coli.. J Mol Biol.

[28] Katoh E, Hatta T, Shindo H, Ishii Y, Yamada H (2000). High precision NMR structure of YhhP, a novel Escherichia coli protein implicated in cell division.. J Mol Biol.

[29] Waterman D. G, Ortiz-Lombardia M, Fogg M. J, Koonin E. V, Antson A. A (2006). Crystal structure of Bacillus anthracis ThiI, a tRNA-modifying enzyme containing the predicted RNA-binding THUMP domain.. J Mol Biol.

[30] Ramelot T. A, Cort J. R, Goldsmith-Fischman S, Kornhaber G. J, Xiao R (2004). Solution NMR structure of the iron-sulfur cluster assembly protein U (IscU) with zinc bound at the active site.. J Mol Biol.

[31] Shimomura Y, Wada K, Fukuyama K, Takahashi Y (2008). The asymmetric trimeric architecture of [2Fe-2S] IscU: implications for its scaffolding during iron-sulfur cluster biosynthesis.. J Mol Biol.

[32] Bordo D, Deriu D, Colnaghi R, Carpen A, Pagani S (2000). The crystal structure of a sulfurtransferase from Azotobacter vinelandii highlights the evolutionary relationship between the rhodanese and phosphatase enzyme families.. J Mol Biol.

[33] Musco G, Stier G, Kolmerer B, Adinolfi S, Martin S (2000). Towards a structural understanding of Friedreich's ataxia: the solution structure of frataxin.. Structure.

[34] Dhe-Paganon S, Shigeta R, Chi Y. I, Ristow M, Shoelson S. E (2000). Crystal structure of human frataxin.. J Biol Chem.

[35] Cho S. J, Lee M. G, Yang J. K, Lee J. Y, Song H. K (2000). Crystal structure of Escherichia coli CyaY protein reveals a previously unidentified fold for the evolutionarily conserved frataxin family.. Proc Natl Acad Sci U S A.

[36] Nair M, Adinolfi S, Pastore C, Kelly G, Temussi P (2004). Solution structure of the bacterial frataxin ortholog, CyaY: mapping the iron binding sites.. Structure.

[37] Shimomura Y, Takahashi Y, Kakuta Y, Fukuyama K (2005). Crystal structure of Escherichia coli YfhJ protein, a member of the ISC machinery involved in assembly of iron-sulfur clusters.. Proteins.

[38] Raulfs E. C, O'Carroll I. P, Dos Santos P. C, Unciuleac M. C, Dean D. R (2008). In vivo iron-sulfur cluster formation.. Proc Natl Acad Sci U S A.

[39] Urbina H. D, Silberg J. J, Hoff K. G, Vickery L. E (2001). Transfer of sulfur from IscS to IscU during Fe/S cluster assembly.. J Biol Chem.

[40] Landau M, Mayrose I, Rosenberg Y, Glaser F, Martz E (2005). ConSurf 2005: the projection of evolutionary conservation scores of residues on protein structures.. Nucleic Acids Res.

[41] Mueller E. G, Palenchar P. M, Buck C. J (2001). The role of the cysteine residues of ThiI in the generation of 4-thiouridine in tRNA.. J Biol Chem.

[42] Wright C. M, Christman G. D, Snellinger A. M, Johnston M. V, Mueller E. G (2006). Direct evidence for enzyme persulfide and disulfide intermediates during 4-thiouridine biosynthesis.. Chem Commun (Camb).

[43] Nilsson K, Lundgren H. K, Hagervall T. G, Bjork G. R (2002). The cysteine desulfurase IscS is required for synthesis of all five thiolated nucleosides present in tRNA from Salmonella enterica serovar typhimurium.. J Bacteriol.

[44] Lauhon C. T, Skovran E, Urbina H. D, Downs D. M, Vickery L. E (2004). Substitutions in an active site loop of Escherichia coli IscS result in specific defects in Fe-S cluster and thionucleoside biosynthesis in vivo.. J Biol Chem.

[45] Zheng L, White R. H, Cash V. L, Dean D. R (1994). Mechanism for the desulfurization of L-cysteine catalyzed by the nifS gene product.. Biochemistry.

[46] Fujii T, Maeda M, Mihara H, Kurihara T, Esaki N (2000). Structure of a NifS homologue: X-ray structure analysis of CsdB, an Escherichia coli counterpart of mammalian selenocysteine lyase.. Biochemistry.

[47] Mihara H, Fujii T, Kato S, Kurihara T, Hata Y (2002). Structure of external aldimine of Escherichia coli CsdB, an IscS/NifS homolog: implications for its specificity toward selenocysteine.. J Biochem.

[48] Tirupati B, Vey J. L, Drennan C. L, Bollinger J. M (2004). Kinetic and structural characterization of Slr0077/SufS, the essential cysteine desulfurase from Synechocystis sp. PCC 6803.. Biochemistry.

[49] Lima C. D (2002). Analysis of the E. coli NifS CsdB protein at 2.0 A reveals the structural basis for perselenide and persulfide intermediate formation.. J Mol Biol.

[50] Smith A. D, Frazzon J, Dean D. R, Johnson M. K (2005). Role of conserved cysteines in mediating sulfur transfer from IscS to IscU.. FEBS Lett.

[51] Kato S, Mihara H, Kurihara T, Takahashi Y, Tokumoto U (2002). Cys-328 of IscS and Cys-63 of IscU are the sites of disulfide bridge formation in a covalently bound IscS/IscU complex: implications for the mechanism of iron-sulfur cluster assembly.. Proc Natl Acad Sci U S A.

[52] Cupp-Vickery J. R, Peterson J. C, Ta D. T, Vickery L. E (2004). Crystal structure of the molecular chaperone HscA substrate binding domain complexed with the IscU recognition peptide ELPPVKIHC.. J Mol Biol.

[53] Kim J. H, Fuzery A. K, Tonelli M, Ta D. T, Westler W. M (2009). Structure and dynamics of the iron-sulfur cluster assembly scaffold protein IscU and its interaction with the cochaperone HscB.. Biochemistry.

[54] Hoff K. G, Silberg J. J, Vickery L. E (2000). Interaction of the iron-sulfur cluster assembly protein IscU with the Hsc66/Hsc20 molecular chaperone system of Escherichia coli.. Proc Natl Acad Sci U S A.

[55] Perna N. T, Plunkett G. I, Blattner F. R, Mau B, Blattner F. R (2001). Genome sequence of enterohaemorrhagic *Escherichia coli* O157:H7.. Nature.

[56] Matthews B. W (1968). Solvent content of protein crystals.. J Mol Biol.

[57] Otwinowski Z, Minor W (1997). Processing of X-ray diffraction data collected in oscillation mode.. Methods Enzymol.

[58] Storoni L. C, McCoy A. J, Read R. J (2004). Likelihood-enhanced fast rotation functions.. Acta Crystallogr D Biol Crystallogr.

[59] Murshudov G. N, Vagin A. A, Dodson E. J (1997). Refinement of macromolecular structures by the maximum-likelihood method.. Acta Crystallogr.

[60] Adams P. D, Afonine P. V, Bunkoczi G, Chen V. B, Davis I. W (2010). PHENIX: a comprehensive Python-based system for macromolecular structure solution.. Acta Crystallographica Section D.

[61] Emsley P, Cowtan K (2004). Coot: model-building tools for molecular graphics.. Acta Crystallogr.

[62] Laskowski R. A, MacArthur M. W, Moss D. S, Thornton J. M (1993). PROCHECK: a program to check the stereochemical quality of protein structures.. J Appl Crystallogr.

[63] Datsenko K. A, Wanner B. L (2000). One-step inactivation of chromosomal genes in Escherichia coli K-12 using PCR products.. Proc Natl Acad Sci U S A.

[64] Martinez-Vicente M, Yim L, Villarroya M, Mellado M, Perez-Paya E (2005). Effects of mutagenesis in the switch I region and conserved arginines of Escherichia coli MnmE protein, a GTPase involved in tRNA modification.. J Biol Chem.

[65] Gehrke C. W, Kuo K. C (1989). Ribonucleoside analysis by reversed-phase high-performance liquid chromatography.. J Chromatogr.

