# Supplementary material for: Structural Basis for Fe–S Cluster Assembly and tRNA Thiolation Mediated by IscS Protein–Protein Interactions
Source: PLoS Biol. 2010 Apr 13;8(4):e1000354. doi: 10.1371/journal.pbio.1000354 (PMC2854127; doi:10.1371/journal.pbio.1000354)
Supplement: Table S1 — Oligonucleotides used for construction of iscS::kan (IC6087) and tusA::kan (IC6085) strains. (0.04 MB DOC) [file pbio.1000354.s009.doc]

# Supplementary Data

# Structural Basis for Fe-S Cluster Assembly and tRNA Thiolation Mediated by IscS Protein-Protein Interactions

Rong Shi^1^, Ariane Proteau^1^, Magda Villarroya^2^, Ismaïl Moukadiri^2^, Linhua Zhang^3^, Jean-Francois Trempe^1^, Allan Matte^3^, M. Eugenia Armengod^2^, Miroslaw Cygler^1,3^,*

^1^Department of Biochemistry, McGill University, Montréal, Québec, Canada

^2^Laboratorio de Genética Molecular, Centro de Investigación Príncipe Felipe, Avenida Autopista del Saler, 16-3, 46012 Valencia, Spain

^3^Biotechnology Research Institute, NRC, 6100 Royalmount Avenue, Montréal, Québec H4P 2R2, Canada

Table S1: Oligonucleotides used for construction of *iscS::kan* (IC6087) and *tusA::kan* (IC6085) strains

Figure S1. Sequences with secondary structure assignments above, h – α-helix, s – β-strand. Secondary structures are numbered in the middle of strings of sssss or hhhhh.

Figure S2. **a)** Stereoview of the IscS-TusA interface with 2mF_o_-DF_c_ electron density shown at 1.0 σ level. The orientation is the same as in Figure 3a. The electron density for chain A of IscS is colored in blue, TusA in magenta. The electron density for the Cys328 loop in chain B of IscS is shown at 0.7 σ level and colored in green. For clarity the residues are not labeled; **b)** Stereoview of the IscS-IscU interface with 2mF_o_-DF_c_ electron density shown at 0.9 σ level. The orientation is the same as in Figure 3b. The density for IscS is colored in blue, IscU in magenta. For clarity the residues are not labeled. This and other structural figures were prepared with the program PyMol ([www.pymol.org](http://www.pymol.org/)).

Figure S3. Conserved surface residues on IscU. Top: cartoon representation, bottom: molecular surface. The level of conservation of surface residues is marked in shades of burgundy (dark – high conservation, white – highly variable).

Figure S4. Superposition of Iscs-TusA and IscS-IscU showing the steric overlap of TusA and IscU. The IscS subunits are painted green and cyan, TusA is magenta and IscU is wheat. The IscS Cys328 loop is colored red and the cysteines in all molecules are shown explicitly in stick mode. The steric clashes would occur in the circled region. The separation between positions of acceptor cysteines of TusA and IscU is in excess of 16 Ǻ and is marked with an arrow. The distance between the cysteines of IscS and TusA is ~4 Ǻ, while the distance between the cysteines of IscS and IscU is greater than 12 Ǻ.

Figure S5. Pull-downs of His-IscS by **a)** IscU mutants and **b)** TusA mutants.

Figure S6. Three-way pull-downs. **a)** Competition between TusA and IscU for IscS. **Left:** The preformed complex of His-IscS/GST-TusA was loaded on Glutathione Sepaharose 4B column (lane 1) and washed thoroughly. Beads incubated with TEV protease and released His_IscS-TusA complex eluted (lane 2). GST-IscU (lane 3) loaded on Glutathione Sepaharose 4B column and washed (lane 4), His-IscS-TusA added and column washed. Incubation with TEV protease and elution of released proteins (lane 5). Only His-IscS and IscU were observed. **Right:** Similar experiment performed in opposite order. First His-IscS-IscU complex was formed and it was loaded on the column with bound GST-TusA. His-IscS-IscU did not bind to the column and only GST-TusA was found on the beads.; **b)** Competition between IscU and CyaY for IscS. The preformed complex of His-IscS/GST-IscU was loaded on Glutathione Sepaharose 4B column and washed thoroughly (lane 1). Since both proteins run at the same place on the SDS-PAGE, the beads were incubated with TEV protease and released proteins were eluted from the column (lane 2) confirming the presence of His-IscS-IscU complex. GST-CyaY was loaded on glutathione column (lane 3) and His-IscS-IscU added to the column and washed (lane 4). Finally, incubation with TEV protease released His-IscS, IscU and CyaY, showing that they formed a ternary complex; **c)** Formation of a ternary complex between His-IscS, IscU and IscX. Similar procedure as in above**; d)** Competition betweenTusA and IscX for IscS. Only His-IscS and IscX eluted after second TEV cleavage; **e)** Competition between TusA and CyaY for IscS. Only His-IscS and CyaY eluted after second TEV cleavage.

Figure S7. Models of the IscS-CyaY complex with restraints from NMR (CyaY) and mutagenesis (IscS). The location of IscU (wheat) relative to IscS is also shown.

Figure S8. Superposition of IscS and SufS. The conformation of the loop bearing the active site cysteine is significantly different in both proteins.

Table S1

| Primer | Sequence^1^ |
| --- | --- |
| TusA (F) | ATGACCGATCTCTTTTCCAGCCCTGACCACACACTCGACGTGTAGGCTGGAGCTGCTTCG |
| TusA (R) | GCCTTTACGAATCAAATAACGATAAGGCAGTCCATCCGTCCATATGAATATCCTCCTTAG |
| IscS (F) | CGACTACTCCGCAACCACGCCGGTGGACCCGCGTGTTGCCTGTAGGCTGGAGCTGCTTCG |
| IscS (R) | CACAGCGGAGAAAGGTCACGCAGACGACCGATGGATTTACCATATGAATATCCTCCTTAG |

^1^Sequences of the *kan* gene are underlined
